# Inactivity of Peptidase ClpP Causes Primary Accumulation of Mitochondrial Disaggregase ClpX with Its Interacting Nucleoid Proteins, and of mtDNA

**DOI:** 10.3390/cells10123354

**Published:** 2021-11-29

**Authors:** Jana Key, Sylvia Torres-Odio, Nina C. Bach, Suzana Gispert, Gabriele Koepf, Marina Reichlmeir, A. Phillip West, Holger Prokisch, Peter Freisinger, William G. Newman, Stavit Shalev, Stephan A. Sieber, Ilka Wittig, Georg Auburger

**Affiliations:** 1 Experimental Neurology, Faculty of Medicine, Goethe University, 60590 Frankfurt, Germany; jana.key88@gmail.com (J.K.); torres.odio@exchange.tamu.edu (S.T.-O.); Gispert-Sanchez@em.uni-frankfurt.de (S.G.); Gabriele.Koepf@kgu.de (G.K.); Marina.Reichlmeir@gmail.com (M.R.); 2 Department of Microbial Pathogenesis and Immunology, College of Medicine, Health Science Center, Texas A&M University, Bryan, TX 77807, USA; awest@tamu.edu; 3 Center for Protein Assemblies, Chair of Organic Chemistry II, Technical University of Munich, Ernst-Otto-Fischer-Strasse 8, 85748 Garching, Germany; nina.bach@tum.de (N.C.B.); stephan.sieber@tum.de (S.A.S.); 4 Faculty of Biosciences, Geothe University, Altenhöferallee 1, 60438 Frankfurt am Main, Germany; 5 Institute of Neurogenomics, Helmholtz Zentrum München, 85764 Neuherberg, Germany; prokisch@helmholtz-muenchen.de; 6 Department of Paediatrics, Kreisklinikum Reutlingen, 72764 Reutlingen, Germany; Freisinger_P@klin-rt.de; 7 Division of Evolution Infection and Genomics, School of Biological Sciences, Faculty of Biology, Medicine and Health, University of Manchester, Manchester M13 9PL, UK; William.newman@manchester.ac.uk; 8 Manchester Centre for Genomic Medicine, St Mary’s Hospital, Manchester University NHS Foundation Trust, Manchester M13 9PL, UK; 9 Genetic Institute, Emek Medical Center, Afula, Rappaport Faculty of Medicine, Technion—Israel Institute of Technology, Haifa 3200003, Israel; stavit_sh@clalit.org.il; 10 Functional Proteomics, Institute for Cardiovascular Physiology, Medical Faculty, Goethe University, 60596 Frankfurt am Main, Germany; wittig@med.uni-frankfurt.de; 11 German Center for Cardiovascular Research (DZHK), Partner Site RheinMain, 60596 Frankfurt am Main, Germany

**Keywords:** leukodystrophy, ataxia, Parkinson’s disease, HARS2, LARS2, TWNK, ERAL1, ClpB

## Abstract

Biallelic pathogenic variants in *CLPP*, encoding mitochondrial matrix peptidase ClpP, cause a rare autosomal recessive condition, Perrault syndrome type 3 (PRLTS3). It is characterized by primary ovarian insufficiency and early sensorineural hearing loss, often associated with progressive neurological deficits. Mouse models showed that accumulations of (i) its main protein interactor, the substrate-selecting AAA+ ATPase ClpX, (ii) mitoribosomes, and (iii) mtDNA nucleoids are the main cellular consequences of ClpP absence. However, the sequence of these events and their validity in human remain unclear. Here, we studied global proteome profiles to define ClpP substrates among mitochondrial ClpX interactors, which accumulated consistently in ClpP-null mouse embryonal fibroblasts and brains. Validation work included novel ClpP-mutant patient fibroblast proteomics. ClpX co-accumulated in mitochondria with the nucleoid component POLDIP2, the mitochondrial poly(A) mRNA granule element LRPPRC, and tRNA processing factor GFM1 (in mouse, also GRSF1). Only in mouse did accumulated ClpX, GFM1, and GRSF1 appear in nuclear fractions. Mitoribosomal accumulation was minor. Consistent accumulations in murine and human fibroblasts also affected multimerizing factors not known as ClpX interactors, namely, OAT, ASS1, ACADVL, STOM, PRDX3, PC, MUT, ALDH2, PMPCB, UQCRC2, and ACADSB, but the impact on downstream metabolites was marginal. Our data demonstrate the primary impact of ClpXP on the assembly of proteins with nucleic acids and show nucleoid enlargement in human as a key consequence.

## 1. Introduction

Primary ovarian insufficiency combined with early-onset sensorineural hearing loss, inherited in an autosomal recessive manner, is the clinical hallmark of Perrault syndrome (PRLTS). As underlying genetic causes, biallelic variants in the genes *CLPP*, *HARS2*, *LARS2*, *TWNK, ERAL1*, *GGPS1*, *PRORP*, and *HSD17B4* have been identified in 50% of cases, and other putative disease genes are under investigation [1,2].

For accurate diagnosis and preventive therapy, it is important to understand what subcellular compartments and pathways are disrupted in each genetic subtype of PRLTS, and what crucial events are in common that lead to the selective pathology of Perrault syndrome. The disease-associated genes mostly encode mitochondrial proteins, with *HARS2*, *LARS2*, and *ERAL1* serving roles for mitoribosomal translation apparatus assembly [3], while *PRORP*, *HARS2*, and *LARS2* jointly function in tRNA processing of mitochondria [4]. The *TWNK* gene product Twinkle is well characterized as a helicase and primase for mitochondrial DNA, and mitochondrial RNA granules were recently found to need it [5], but no mitoribosomal function has been described to date. Interestingly, one Twinkle isoform is targeted to the nucleus and localizes to the kinetochore during progenitor cell division [6], a fact that might relate to the primary oocyte failure in Perrault syndrome. An apparently unrelated localization and function is known for the *GGPS1* gene product Geranylgeranyl Diphosphate Synthase 1, a cytosolic key enzyme for carotenoid biosynthesis, for the C20 prenylation of proteins, and for the regulation of a nuclear hormone receptor [7].

A wider spectrum of targets was described for the mitochondrial matrix peptidase ClpP, which plays important roles during the unfolded protein response for mitochondria (UPR^mt^) [8,9]. It depends on AAA+ ATPases such as ClpX for activation, substrate selection, and disaggregation, thus converting into a protease [10,11,12]. Two heptameric ClpP rings assemble with hexameric ClpX rings on either side into a barrel-shaped unfolding and degradation machine [13]. In contrast, ClpB, as another member of the Clp/Hsp100 AAA+ ATPase family, acts to disaggregate proteins in concert with the cognate DnaK/Hsp70 chaperone system, without associating with ClpP or activating degradation [14]. Homozygous deletion of ClpP in mouse was found to trigger accumulated levels of the disaggregase ClpX, mitoribosomes, and mitochondrial nucleoids [15,16,17], but it remains unclear whether this pathology exists in human and which event is primary. Whether the ClpP-null-triggered, on average 1.3-fold accumulation of mitoribosomal proteins depends on the RNA chaperone ERAL1 or other factors also remains under debate [16,18]. ClpP deficiency in mouse also causes reduced body size with leanness, even before the manifestation of complete infertility and hearing impairment [15,19,20]. Interestingly, ClpP-mutant mice and patients affected by Perrault syndrome type 3 (PRLTS3) display not only primary ovarian insufficiency, as in other forms of PRLTS, but also azoospermia [15,21], indicating that germ cell differentiation and meiosis have profound defects, while mitotic proliferation rates of ClpP-null mouse cells appear normal. The PRLTS3 infertility is complete, a phenotype much stronger than the asthenozoospermia usually observed in mitochondrial disorders [22], suggesting that severe extra-mitochondrial pathology may contribute to PRLTS3.

The existence of retrograde signaling about mitochondrial needs and problems to the nucleus has been well established; in particular, the communication via calcium release and endoplasmic reticulum signals are well understood [23]. How UPR^mt^ is sensed and executed in the nucleus can apparently be mediated by a host of differing mechanisms across phyla [24]. In the age-associated neurodegenerative disorder known as Parkinson’s disease, pathogenic variants of the mitochondrial membrane factor CHCHD2 were shown to stimulate UPR^mt^ via CHCHD2 nuclear relocalization and via CHCHD10 oligomerization [25,26,27]. As a main nuclear transcriptional response to ClpP-null-associated mitochondrial pathology in mice, a massive induction of innate immunity factors was documented, triggered by mtDNA and mitoribosomal stress via the cGAS-STING signaling pathway, and by the nuclear accumulation of the co-chaperone DNAJA3 [17,28,29,30]. Again, it is unclear if such neuroinflammatory events underlie the PRLTS3 neurodegenerative process in humans.

**Figure 1:**
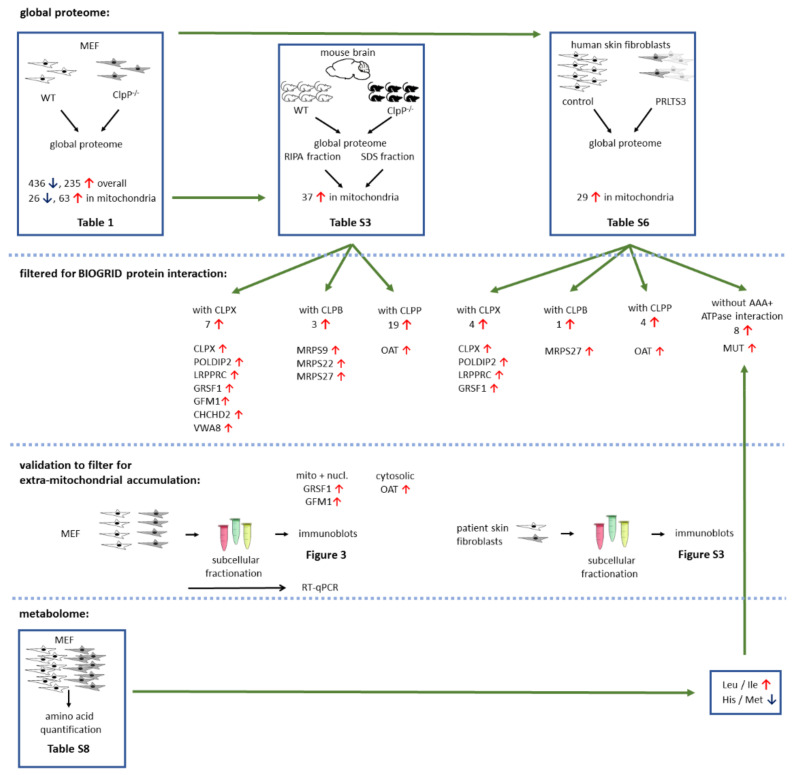
Schematic overview of experimental setup and main findings. Numbers represent amount of dysregulated factors, red arrows pointing up indicate significant upregulations, and blue arrows pointing down indicate downregulations. Number of biological replicates (different cell lines or mice) is represented by non-overlapping symbols, and cell line triplicates are shown as overlapping.

Here, the identification of initial events of pathogenesis was achieved by a multi-layered approach: The consistency of ClpP-dependent protein accumulations was assessed in global proteome profiles from mouse embryonal fibroblasts (MEFs), mouse brain fractions, and skin fibroblasts from patients with biallelic ClpP variants. This approach was conducted under the assumption that the comparison of fibroblasts (where energy is mainly generated by glycolysis and the downstream mitochondrial Krebs cycle) versus brain tissue (where energy is mainly generated via mitochondrial respiration) would help to define the invariant key ClpXP substrates. We classified accumulated mitochondrial proteins according to evidence if (i) they are bona fide interactors of the disaggregase ClpX, (ii) targets of the disaggregase ClpB, (iii) protein interactors of ClpP itself, or (iv) rather may represent indirect effects due to delayed protein complex turnover and inefficient assembly that are consequences of altered chaperone abundance. The degree to which such misassembly of matrix enzymes impacts mitochondrial function was studied by mouse fibroblast metabolome mass spectrometry and from a literature review (scheme in Figure 1). Immunoblots of putative ClpP substrates in subcellular fractions assessed if excess amounts of ClpX and its interactors associate with docking sites outside mitochondria. The findings provide prominent novel insights into how ClpP inactivity has an early preferential ClpX-dependent impact on tRNA processing factors of mitochondria (and possibly the nucleus). As variants in other tRNA processing factors such as PRORP, HARS2, and LARS2 are a known cause of PRLTS, a common mechanism of disease pathogenesis begins to emerge for Perrault syndrome. In addition, consistent accumulations that are potentially independent of ClpX were observed for specific vitamin-associated multimerized factors in the mitochondrial matrix.

## 2. Materials and Methods

### 2.1. Mice

ClpP-null mice had been generated previously [15], were bred from heterozygous matings, and were housed under specific-pathogen-free conditions with monthly testing of sentinel animals, using a 12 h light cycle with food and water *ad libitum* in the central animal facility (ZFE) at Frankfurt University Hospital. All animal experiments were performed in compliance with the German animal welfare law.

### 2.2. MEF Generation and Culture

Generation and cell culture of mouse embryonic fibroblasts (MEFs) were conducted as previously described [29]. After intercrosses of ClpP^+/−^ mice, WT and ClpP^−/−^ MEFs were prepared from embryos at 14.5 days *post-coitus*. Cells were grown in Dulbecco’s modified Eagle medium (DMEM) (Gibco, Thermo Fisher Scientific, Waltham, MA, USA) supplemented with 1% L-glutamine (Gibco, Thermo Fisher Scientific) and 10% fetal bovine serum (FBS) (Gibco, Thermo Fisher Scientific) in a humidified incubator at 37 °C and 5% CO_2_.

### 2.3. Label-Free Quantification of Proteins in Wild-Type and ClpP-Null Mouse Embryonic Fibroblasts

The mass spectrometry proteomics data were deposited in the ProteomeXchange Consortium (http://www.proteomexchange.org/, last accessed on 26 November 2021) via the PRIDE [31] partner repository with the dataset identifier PXD023677. Details of sample preparation, quantitative mass spectrometry, and protein identification/quantification are also available in PXD023677. Identifications from the reverse decoy database, by site and known contaminants, were excluded. Data were further bioinformatically analyzed by Perseus 1.5.2.6, (Max-Planck-Institute for Biochemistry, 80539 München, Germany) [32] and Microsoft Excel, (version 2016, Redmont, WA, USA). For quantification, proteins were quality filtered according to a minimum of 3 valid values in 1 group (*n* = 3). All missing values from this reduced matrix were replaced by background values from a normal distribution. For statistical comparison, Student’s *t*-tests were used. Quantification results are summarized in tables. Data were visualized by volcano plots to highlight the most significant hits. Heatmaps, volcano plots, and correlation profiles were generated by Perseus 1.5.2.6.

### 2.4. Sequential Proteome Analysis of Clp^−/−^ versus WT Mouse Brain Tissue

Female 15-month-old WT and age-matched ClpP^−/−^ (*n* = 6 versus 6) mice were sacrificed by decapitation. Their brains were dissected and snap frozen in liquid nitrogen. Frozen brain samples were homogenized in ice-cold buffer (150 mM NaCl, 0.5 mM EDTA, 100 mM Tris pH 7.4) using a motor-driven Potter–Elvehjem with 15 strokes. Samples were further sonified with 10 pulses. Following centrifugation at 16,000 rpm for 10 min at 4 °C, the supernatant was transferred to a new tube and precipitated with 20% TCA. The pellet was resolved in extraction buffer (10% SDS, 150 mM NaCl, 50 mM HEPES pH 7.8). Precipitated proteins were washed twice with ice-cold acetone and finally resolved in extraction buffer. Sonification for 5 s facilitated resolubilization of proteins. An amount of 100 µg of protein from each fraction was diluted in 4% (*w*/*v*) SDS, 100 mM HEPES, pH 7.6, 150 mM NaCl, 0.1 M DTT, mixed with 200 µL 8 M Urea, 50 mM Tris/HCl, pH 8.5, and loaded to spin filters with a 30 kDa cut-off (Microcon, Burlington, MA, United States). The filter-aided sample preparation protocol (FASP) [33] was essentially followed. Proteins were digested overnight with trypsin/LysC (sequencing grade, Promega, Madison, WI, United States). Following established protocols [34], acidified peptides (final concentration 0.1% *v/v* trifluoroacetic acid) were fractionated on multi-stop-and-go tips (StageTips, Thermo Fisher Scientific, Waltham, MA, USA) containing C18 tips and strong cation exchange (SCX) tips. Peptides from the pellet fraction were eluted in 3 steps. The C18 translution fraction was combined with the first SXC fraction as well as the second and third SCX fractions. Peptides from the supernatant fraction were eluted in 6 steps. All fractions of each sample were eluted in wells of microtiter plates. Peptides were dried and resolved in 1% acetonitrile, 0.1% formic acid.

Liquid chromatography/mass spectrometry (LC/MS) was performed on a Thermo Scientific™ Q Exactive Plus equipped with an ultra-high performance liquid chromato-graphy unit (Thermo Scientific EASY- nLC) and a Nanospray Flex Ion-Source (Thermo Scientific, Waltham, MA, USA). Peptides were loaded on a C18 reversed-phase precolumn (Thermo Scientific) followed by separation on a 2.4 µm Reprosil C18 resin (Dr. Maisch GmbH, Ammerbuch, Germany) in-house packed picotip emitter tip (diameter 75 µm, 30 cm long from New Objectives, Aarle-Rixtel, Netherlands) using a gradient from mobile phase A (4% acetonitrile, 0.1% formic acid) to 30% mobile phase B (80% acetonitrile, 0.1% formic acid) for 75 min followed by a second gradient to 60% B for another 30 min with a flow rate 250 nL/min. The run was finished by column washout with 99% B for 5 min and re-equilibration in 1% B.

Xcalibur Raw files were analyzed by proteomics software Max Quant (1.5.3.30, München, Germany) [35]. The enzyme specificity was set to Trypsin, and missed cleavages were limited to 2. Acetylation of the N-terminus (+42.01) and oxidation of methionine (+15.99) were selected for variable modification, and carbamidomethylation (+57.02) on cysteines was set as a fixed modification. The mouse proteome set from Uniprot (Download 26 February 2016) was used to identify peptides and proteins. The false discovery rate (FDR) was set to 1%. Label-free quantification values were obtained from at least one identified peptide. Identifications from the reverse decoy database, by site and known contaminants, were excluded. Data were further bioinformatically analyzed by Perseus 1.5.2.6. (http://coxdocs.org/doku.php?id=perseus:start, accessed on 8 March 2021) and Microsoft Excel. For quantification, proteins were quality filtered according to a minimum of five valid values in one group (while total sample number *n* = 6). All missing values from this reduced matrix were replaced by background values from a normal distribution. For statistical comparison, Student’s *t*-tests were used. Quantification results are summarized in tables. Data were visualized by volcano plots to highlight the most significant hits. Heatmaps, volcano plots, and correlation profiles were generated by Perseus 1.5.2.6. Pathway analysis was conducted by DAVID Bioinformatics Resources 6.7 (Frederick, MD, USA), GOrilla GO [36], and KEGG (Kyoto Encyclopedia of Genes and Genomes) pathway analysis.

### 2.5. Metabolome

For the quantification of 54 metabolic compounds in MEFs, 100 mg cell pellets (1 × 10^7^ cells per line, for 8 WT versus 8 ClpP^−/−^) were snap frozen in liquid nitrogen, stored at −80 °C, and shipped on dry ice to the company Metabolomic Discoveries (Potsdam, Germany). Targeted profiling with unambiguous characterization and relative quantification by LC-tandem mass spectrometry was performed on a Shimadzu (Kyoto, Japan) triple quadrupole LCMS-8050 equipped with an electrospray ionization (ESI) source and operated in multiple reaction mode (MRM). For statistical evaluation, raw data as peak abundances normalized to internal standards and, if necessary, protein content were detailed, complemented by means and standard deviation values, as well as a differential analysis sheet with ANOVA across all groups as a global *p*-value, and an adjusted global *p*-value, followed by a local *p*-value from pairwise *t*-tests and ratios of the group means as a log_2_ value, as well as absolute fold changes. Absolute numbers were normalized against the mean of WT samples. Bar graphs were created with GraphPad Prism (GraphPad Software, San Diego, CA, USA) version 9.

### 2.6. Quantitative Immunoblots

Protein was isolated with RIPA buffer (50 M TRIS/HCl pH 8.0, 150 mM NaCl, 0.1% SDS, 1% triton, 0.5% sodium deoxycholate, 2 mM EDTA, protease inhibitor cocktail (Sigma Aldrich, St. Louis, MI, USA)) as previously described [15]. Protein content was determined using BCA assay (Life Technologies, Carlsbad, CA, USA). An amount of 15 µg of protein was loaded for quantitative immunoblotting. Subcellular fractions were done as previously published [29]. Antibodies used were against HSP60 (SantaCruz (Dallas, TX, USA) sc-13115; 1:500), Lamin A/C (Abcam (Cambridge, UK), ab169532; 1:1000), GAPDH (Calbiochem (Merck Millipore, Darmstadt, Germany), CB1001; 1:1000), CLPX (murine: Invitrogen (Waltham, MA, USA), PA5-79052; 1:1000; human: Sigma Aldrich, HPA040262; 1:1000), POLDIP2 (Proteintech (Manchester, UK), 15080-1-AP; 1:1000), LRPPRC (Invitrogen, PA5-22034; 1:1000), GRSF1 (Sigma Aldrich, HPA036985; 1:1000), GFM1 (Proteintech, 14274-1-AP; 1:1000), CHCHD2 (Proteintech, 19424-1-AP; 1:1000), OAT (Invitrogen, PA5-92842; 1:1000), and MUT (Proteintech, 17034-1-AP; 1:1000).

### 2.7. Human Primary Skin Fibroblasts

Skin cells were donated by a 33-year-old male Arabic individual with a homozygous ClpP mutation (c.[430T>C]; p.[Cys144Arg]; ID-number CB16-0006) who was diagnosed with bilateral sensorineural hearing loss at 14 months of age [21]. The deafness has continuously progressed since. He developed foot drop at 24 years of age with sensory-motor demyelinating axonal peripheral neuropathy of the lower limbs upon neurophysiological testing. In adulthood, he was shown to have azoospermia.

Further skin cells became available from a 4-year-old female Turkish individual with a homozygous ClpP mutation (c.[661G>A]; ID-number 58955) leading to open reading frameshift and undetectable ClpP protein in fibroblast immunoblots [37]. This patient in the first week of life already had muscular weakness. At age 2 months, she was diagnosed with microcephaly (1 cm < 3rd percentile) and showed generalized muscular hypotonia. Echocardiography demonstrated mild hypertrophic cardiomyopathy. Metabolic analysis disclosed repeatedly metabolic acidosis with elevated lactate levels (2.8 to 9.0 mmol/L, normal <2.3 mmol/L). Fumaric acid, 2-oxo-glutaric acid, and methylmalonic acid were mildly elevated in her urine. Respiratory complex III and IV activity were reduced in muscle biopsy [37]. Subsequently, progressive developmental delay and deafness became evident. She developed epilepsy (West syndrome). At the time of skin biopsy, she was 5 years of age.

For comparison, primary skin fibroblast cultures from the Coriell depository (lines AG06103, AG02261, and AG06858) and other cultures from healthy controls kindly provided by Prof. Henry Houlden (UCLH, London, UK) (4 cases) were studied. Cell lines were cultivated in DMEM (Gibco, Thermo Fisher Scientific) supplemented with 1% l-glutamine (Gibco) and 10% fetal bovine serum (FBS) (Gibco, Waltham, MA, USA) in a humidified incubator at 37 °C and 5% CO_2_. This study was conducted according to the guidelines of the Declaration of Helsinki and approved by the Ethics Committee of Klinikum Goethe Universität Frankfurt/Main (protocol code 117/07, 24 May 2007) and the Wales Research Ethics Committee (protocol code 16/WA/0017 and original approval, 4 February 2016).

### 2.8. Polymerase Chain Reaction (PCR) to Control for Mycoplasma Contamination

Here, 200 µL cell culture media from cultures that had grown for at least 48 h were collected and boiled for 5 min at 95 °C. Samples were centrifuged for 2 min at maximum speed. The PCR reaction mix contained 1 µL sample, 7.4 µL water, 0.8 µL primer forward, 0.8 µL primer reverse, and 10 µL HotStarTaq DNA Polymerase (Qiagen, Hilden, Germany).

Forward primers contained

Myco-5-1 CGCCTGAGTAGTACGTTCGC,
Myco-5-2 CGCCTGAGTAGTACGTACGC,
Myco-5-3 TGCCTGAGTAGTACATTCGC,
Myco-5-4 TGCCTGGGTAGTACATTCGC,
Myco-5-5 CGCCTGGGTAGTACATTCGC,
Myco-5-6 CGCCTGAGTAGTATGCTCGC,

and reverse primers contained

Myco-3-1 GCGGTGTGTACAAGACCCGA,
Myco-3-2 GCGGTGTGTACAAAACCCGA,
Myco-3-3 GCGGTGTGTACAAACCCCGA.

Each primer was dissolved to a final concentration of 100 µM, and forward and reverse were mixed to a final concentration of 10 µM each. PCR was conducted at the following times and temperatures: 95 °C for 5 min, (95 °C for 15 s, 56 °C for 15 s, 72 °C for 30 s) × 40, 72 °C for 3 min. Samples were run on a 1.5% agarose gel (agarose: Thermo Fisher Scientific).

### 2.9. Global Proteome of Human Fibroblasts

Seven control subject cell lines and the two PRLTS3 patient cell lines (in triplicates) were cultured in T75 flasks, washed with PBS (Gibco), and lysed with trypsin (Gibco). The lysates were centrifuged and resuspended in 150 µL lysis buffer (50 mM Tris pH 7.5, 150 mM NaCl, 1% NP40, 0.1% sodium deoxycholate, 1 mM EDTA, and protease inhibitor). Protein content was determined using BCA assay (Life Technologies). An amount of 300 µg of each sample was precipitated according to the method of Wessel and Flügge [38], and the proteins were resuspended in 8 M Urea for denaturation. Samples were loaded on molecular cut-off spin columns (Microcon 30 kDa, Merck Millipore) and subsequently reduced, alkylated, and digested with trypsin on the filters following the FASP protocol described by Wiśniewski et al. [33]. The obtained peptide mixtures were lyophilized, desalted on 50 mg SepPac C18 columns (Waters), and afterwards fractionated on self-made SCX stage tips (3 disks of SCX material, Empore, Sigma Aldrich, St. Louis, MI, USA, in 200 µL pipette tips). Peptides bound to the SCX material were stepwise eluted with five different concentrations of AcONH4 (20 mM, 50 mM, 100 mM, 250 mM, and 500 mM), 0.1% TFA, 15% acetonitrile. Overall, 65 samples (5 fractions for each of the 13 fibroblast culture samples) were further lyophilized, desalted on self-made C18 stage tips (2 layers of C18 disks, Empore), and, after final lyophilization, resuspended in 25 µL 0.1% formic acid (FA) and filtered through equilibrated 0.2 µm Millipore filters before mass spectrometry (MS) measurement.

Nanoflow liquid chromatography-MS/MS analysis was performed on an Orbitrap Fusion mass spectrometer (Thermo Fisher) coupled to an UltiMate 3000 Nano HPLC system (Thermo Fisher). Peptides were first loaded on a C18 Acclaim PepMap100 trap column (75 µm ID × 2 cm) and then separated on an Aurora UHPLC analytical column, 75 µm ID × 25 cm, 120 Å pore size (Ionopticks, Fitzroy, Australia). Columns were constantly heated at 40 °C. Subsequent separation was performed using a first gradient ranging from 5 to 22% acetonitrile in 0.1% FA for 105 min followed by a second gradient ranging from 22 to 32% acetonitrile in 0.1% FA for 10 min at an overall flow rate of 400 nL/min. Peptides were ionized via electrospray ionization. Measurements on the Orbitrap Fusion were carried out in a top speed data-dependent mode using a cycle time of 3 s. Full scan (MS1) acquisition (scan range of 300–1500 *m*/*z*) was performed in the Orbitrap at a defined resolution of 120,000, with an automatic gain control (AGC) ion target value of 2e5, whereby dynamic exclusion was set to 60 s. For fragmentation, precursors with a charge state of 2–7 and a minimum intensity of 5e3 were selected and isolated in the quadrupole using a window of 1.6 *m*/*z*. Subsequent fragment generation was achieved using higher-energy collisional dissociation (HCD, collision energy: 30%). The MS2 AGC was adjusted to 1e4, and 35 ms was selected as the maximum injection time for the ion trap (with inject ions for all available parallelized times enabled). Scanning of fragments was performed by applying the rapid scan rate.

MS Bioinformatics: MS raw files were analyzed with MaxQuant software (version 2.0.3.0, München, Germany). The search was based on the Uniprot human reference protein database downloaded on 10 October 2021, containing 20,371 entries. Most default settings of MaxQuant were applied: PSM and protein FDR 1%; enzyme specificity trypsin/P; minimal peptide length: 7; variable modifications: methionine oxidation, N-terminal acetylation; fixed modification: carbamidomethylation. For protein identification, the minimal number of unique peptides was set to 2. The match between runs option was enabled. For label-free protein quantification, the MaxLFQ algorithm was used as part of the MaxQuant environment: (LFQ) minimum ratio count: 2; peptides for quantification: unique. Statistical analysis was performed in Perseus (version 1.6.15.0, accessed on 29 September 2021). Proteins identified only by site, reverse hits, or potential contaminants were removed. LFQ intensities were log2 transformed. Samples were grouped into two categories: control (7 samples) and PRLTS3 (3 samples for patient 0006, plus 3 samples for patient 58955), and then filtered for at least four valid values in each group. The replicate groups were compared via a two-sided, two-sample Student’s *t*-test (S0 = 0, permutation-based FDR method with FDR = 0.05 and 250 randomizations).

### 2.10. Immunofluorescence

Human fibroblasts were grown on 12 mm coverslips 16 h before experiments. The next day, cells were washed with PBS, fixed with 4% paraformaldehyde for 20 min, permeabilized with 0.1% Triton X-100 in PBS for 5 min, and blocked with PBS containing 10% FBS for 30 min. After blocking, cells were incubated with primary antibodies for 60 min and stained with secondary antibodies for 60 min. Cells were washed with PBS between each step. Coverslips were mounted with Prolong Gold anti-fade reagent containing DAPI (Molecular Probes, Eugene, OR, USA). Images were collected with a Nikon Eclipse Ti2 inverted microscope using a 60X oil-immersion objective and NIS-Elements Advanced Research software, version 5.21.02 (Amsterdam, The Netherlands). Z-stack images were processed using the NIS Extended Depth of Focus module to create focused, single-plane maximum intensity projection TIFs. Nucleoid area quantification was performed largely as described [17]. Briefly, approximately 3–4 unique fields of view from 3 distinct images (of each genotyping at each treatment) comprising between 800 and 1000 nucleoids were captured at random. Scale information was added to Image J (Bethesda, MD, USA), then images were made binary, and the area of each nucleoid was determined using the ‘Analyze Particles’ feature of ImageJ. Nucleoids were divided into 2 size cut-offs: <150 nm^2^ and ≥150 nm^2^, and values were plotted as percentage (%). The following antibodies were employed: anti-TFAM (Proteintech (Manchester, UK), 23996-1-AP; 1:200), anti-HSP60 (Santa Cruz, (Dallas, TX, USA), sc-1052; 1:300), anti-ClpP (Proteintech (Manchester, UK), 60004-1-LG; 1:200), anti-DNA (Millipore (Darmstadt, Germany), CBL-186 AC-30-10; 1:300).

### 2.11. Quantitative Polymerase Chain Reaction (qPCR)

Total cellular DNA was isolated with 250 µL (12-well plate) of 50 mM NaOH and boiled for 30 min to solubilize DNA. An amount of 25 mL of 1 M Tris-HCl pH 8 was added to neutralize the pH, and DNA was quantified and diluted to obtain a 2 ng/µL concentration. DNA was then subjected to qPCR using Fast SYBR Green Master Mix (Applied Biosystems, Waltham, MA, USA) and primers indicated below. Three technical replicates were performed for each biological sample, and expression values of each replicate were normalized against KIR4 using the 2^−ΔddCt^ method. Results were plotted as relative mtDNA abundance (fold), and control samples were centered at 1. Forward and reverse primer sequences:

For *ND1* gene,

forward GAACTAGTCTCAGGCTTCAACATCG,
reverse CTAGGAAGATTGTAGTGGTGA.

For D-loop,

forward CATAAAGCCTAAATAGCCCACACG,
reverse CCGTGAGTGGTTAATAGGGTGATA.

For *KIR4* gene,

forward GCGCAAAAGCCTCCTCATT,
reverse CCTTCCTTGGTTTGGTGGG.

### 2.12. Reverse Transcriptase Quantitative Real-Time Polymerase Chain Reactions (RT-qPCRs)

Total RNA from snap-frozen cell pellets (*n* = 4 WT versus 4 ClpP^−/−^ MEF lines) was isolated with TRI reagent (Sigma, Burlington, MA, USA). DNase (Amplification Grade, Invitrogen) was used for purification of RNA, and SuperScript III (Invitrogen) for reverse transcription, following manufacturers’ instructions. qPCR was performed with TaqMan Gene Expression Assays (Applied Biosystems) in cDNA from 20 ng total RNA in 20 µL reactions with 2× master mix from Roche in a StepOnePlus Real-Time PCR System (Applied Biosystems, Waltham, MA, USA). The analysis of the data was carried out with the 2^−ΔΔCT^ method [39]. The following Taqman assays (ThermoFisher, Waltham, MA, USA) were employed for murine transcripts: *ClpP*: Mm00489940_m1, *ClpX*: Mm00488586_m1, *Poldip2*: Mm00458936_m1, *Lrpprc*: Mm00511512_m1, *Grsf1*: Mm00618579_m1, *Gfm1*: Mm00506856_m1, *Chchd2*: Mm01742631_s1, *Mut*: Mm00485312_m1, *Oat*: Mm00497544_m1, *Tbp*: Mm00446973_m1.

### 2.13. Bioinformatic Analyses

Global proteome Perseus output data were further analyzed employing the STRING webserver (https://string-db.org/, last accessed on 1 July 2021) for protein–protein interaction and pathway analyses. Filtered gene symbols were entered into the Multiple Proteins option for *Mus musculus*, and the graphical output was archived, highlighting factors with identical features by coloring, as explained in figure captions. Significant enrichments of GO (Gene Ontology) terms and KEGG pathways were exported into Excel files. For further protein interaction studies, the biomedical interaction repository BioGrid (https://thebiogrid.org/, last accessed on 6 September 2021) was used. The interactors for human ClpP, ClpX, and ClpB were searched independently, and the BioGRID output data were saved as Excel files. Data were manually filtered for non-redundant factors in separate work sheets, and proteins were matched with global proteome data. Overlapping proteins between different datasets were identified by creating Venn diagrams using the website http://bioinformatics.psb.ugent.be/webtools/Venn/, last accessed on 11 September 2021. An overview of Venn output data is included in Figure 1. Fisher’s exact test implemented in Perseus was used to identify non-random associations between terms in categorical columns. Tables include *t*-test *p*-values and the Benjamini–Hochberg FDR (multiple hypothesis testing). Statistical evaluation was conducted using GraphPad Prism version 9. Bar graphs show variances as the standard error of the mean (SEM) and *p*-values from Welch’s *t*-test (* *p* < 0.05; ** *p* < 0.01; *** *p* < 0.001; **** *p* < 0.0001).

## 3. Results

### 3.1. ClpP-Null MEF Proteome Profile and Its Bioinformatic Analysis

We aimed to identify ClpP-dependent protein degradation deficits, and to define a sequence of events within mitochondria to elucidate the initial events in PRLTS3 pathogenesis. Primary fibroblasts were prioritized since they are easily available from ClpP-mutant patient skin and from mouse embryos. Cultures of three ClpP-null versus three age-/sex-matched WT MEFs were extracted with 10% SDS and analyzed with label-free mass spectrometry to survey alterations in protein abundance. Among 5838 detected factors (Table S1 complete with imputation), 436 were significantly decreased, while 235 showed significantly increased levels. Preferential mitochondrial localization is known for 26 downregulated and 63 upregulated factors (Table 1). Putative ClpP-dependent degradation substrates would be expected among the latter ones. The absence of ClpP and the previously reported accumulation of its substrate-selecting interactor ClpX [15] were confirmed in the mass spectrometry spectra.

Imputation was performed to maximize the number of proteins under analysis, and the power of subsequent bioinformatics. Evaluation of significant interaction clusters and pathway enrichment among these factors employed the Fisher algorithm. This approach revealed that the accumulations occurred mainly among extra-mitochondrial proteins of the immune system and the extracellular matrix, while accumulated proteins in mitochondria had a smaller number and amount of fold changes (most of them are known for a role in the RNA metabolic process). In comparison, the reduced protein abundance was clustered among nuclear proteins, particularly the spliceosomal complex (Table S1, further datasheets). It was intriguing to note how mitochondrial ClpP inactivity affects nuclear functions, but the mechanisms of this retrograde signaling require further elucidation. The greater fold changes of extra-mitochondrial factors may be due to cytosolic signals being amplified over many steps by orders of magnitude, as is known for phosphorylation and blood coagulation cascades, and the strong inducibility of nuclear promoters (e.g., for immediate-early genes) when there is no need to maintain stoichiometric ratios with mitochondrially encoded proteins. Thus, with largest effect sizes reflecting very downstream events, we filtered by significance levels rather than fold changes in our subsequent quest to identify primary events of pathogenesis.

In a more stringent analysis, we focused on those 169 proteins that were significantly upregulated (*p* < 0.03) upon analysis without imputation. These potential ClpP substrates were assessed on the STRING web platform, revealing a strongly significant protein–protein interaction enrichment (*p* < 1 × 10^−16^). STRING generated a diagram of protein–protein interactions that highlights a prominent network of mitoribosomal components and molecular chaperones, as described [12,16]; in comparison, the assembly factors for iron–sulfur cluster biosynthesis and respiratory chain elements as well as mitochondrial enzymes played a minor role (Figure S1). The detailed analysis of enrichments (Table S2) also documented the previously reported increase in cytosolic innate immunity mediators [15], and a novel elevation in secreted collagen precursors. Overall, the global proteome profiles of ClpP-null MEFs were in excellent agreement with previous knowledge, but further filtering was needed to identify ClpP substrates with a primary role in pathogenesis. It remained unclear how to best eliminate the manifold downstream cell-wide effects.

### 3.2. Selected Dysregulations Are Reproducible in Brain Tissue and Reflect Direct ClpX Impact on Interactors

**Figure 2:**
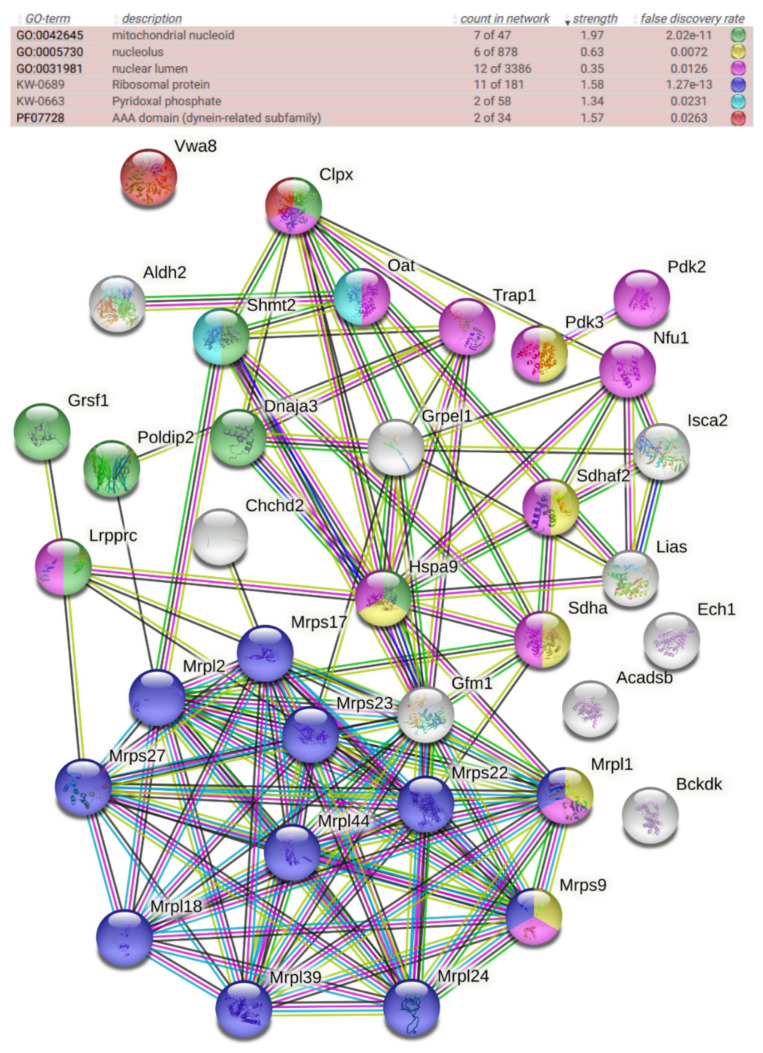
STRING protein–protein interaction diagram of significantly upregulated factors, consistent among the global proteomes of MEFs, brain supernatants, and brain pellet fractions. The color of each factor and the significance of enrichment are detailed above the diagram, showing its implication in functional contexts. The color of lines between factors reflects the degree of known associations.

Thus, we tested if these accumulations also occur in different cell fractions of female 15-month-old ClpP-null mouse brains. At this mouse stage, the cell stress of old age already becomes apparent, while senescent multimorbidity has not yet begun. Sequential extractions from the brain tissue were performed, first obtaining readily solubilized proteins with RIPA buffer, and subsequently solubilizing the resulting pellets in 2% SDS buffer to also study the more insoluble proteins. After label-free mass spectrometric quantification of individual peptides, the two resulting global proteome profiles from the brain extract supernatant and pellet (Table S3) were compared with the initial data from complete MEFs (Table S1). The significantly upregulated factors were filtered for consistency among all lists, and for mitochondrial localization, producing a list of 37 potential ClpP degradation substrates (Table S3). In the resulting STRING diagram (Figure 2) and pathway enrichment statistics (Table S4), prominent accumulations of mitoribosomal proteins (blue color) and chaperones were observed as before, and mitochondrial nucleoid factors became more prominent (green), with most of these diagram nodes connected to ClpX accumulation. Interestingly, several of the consistently upregulated factors localized not only to mitochondria but, in parallel, also to the nucleus, particularly the nucleolus (Figure 2).

To act as a degrading protease, ClpP depends on ClpX for the selection and disaggregation of its substrates, while ClpP, by itself, can only cleave small peptides. Thus, in our reasoning, the accumulation of ClpX and its interactor proteins would be among the initial primary events of pathogenesis in ClpP-mutant cells. To ensure that all available high-quality data on endogenous protein complexes entered this analysis, the manually curated BioGrid database entry on human ClpX protein associations was downloaded (Table S5). Among these known ClpX interactors, the ClpP-null fibroblasts and brain tissues exhibited consistent significant accumulations for the AAA+ ATPase ClpX itself, another AAA+ ATPase in mitochondria known as VWA8 (Von Willebrand Factor A Domain Containing 8) [40], the nucleoid component POLDIP2 (also known as PDIP38) (DNA Polymerase Delta Interacting Protein 2) [41], the mitochondrial RNA granule component LRPPRC (Leucine Rich Pentatricopeptide Repeat Containing) [42], the mitochondrial precursor RNA processing factor GRSF1 (G-Rich RNA Sequence Binding Factor 1) [43], the mitoribosomal tRNA translocation mediator GFM1 (G Elongation Factor Mitochondrial 1) [44], and the UPR^mt^ sensor CHCHD2 (Coiled-Coil-Helix-Coiled-Coil-Helix Domain Containing 2) [25,45].

To assess the specificity of these observations, in comparison, we assessed the protein interaction list for the AAA+ ATPase ClpB, which is also conserved from bacteria to mammalian mitochondria. Among the ClpB interactor proteins listed in BioGrid, only three components of the small mitoribosomal subunit named MRPS9, MRPS22, and MRPS27 showed consistent significantly upregulated abundance in ClpP-null MEFs and brains (Table S5).

In a third BioGrid data mining effort, we looked into ClpP interactor proteins. Significant accumulation in ClpP-null MEFs was observed for 37 such factors, among which 19 were also upregulated in ClpP-null brains (Table S5). Interestingly, only 6 of them are known to associate with ClpP and with ClpX in parallel, but 13, and thus a majority, are documented only as ClpP interactors, but not as ClpX interactors (e.g., OAT, ornithine amino transferase). Naturally, such ClpX interaction may simply have been overlooked by investigators focused on identifying ClpP substrates and might still be documented in the future by more advanced methods. However, the frequency of this phenomenon made us consider what the effects of ClpP activity would be when it is not associated with ClpX. It is known (i) that the stoichiometric ratio between the peptidase ClpP and the disaggregase ClpX is not fixed between different tissues, (ii) that ClpX has some effects that are independent from ClpP [46], and (iii) that the split of the ClpXP proteolytic digestion machinery into two subunits contrasts with LonP1, where the substrate recognition/disaggregase domain and the protease domain coexist within the same subunit (in complete conservation across phyla). Thus, we subsequently assessed the possibility that ClpP deficiency might lead to accumulation of cleavage substrates that do not interact with ClpX.

Overall, the additional proteomic work to define dysregulation consistency between glycolytic fibroblasts and respiring brains, together with the filtering of dysregulated ClpX protein interactors, permitted us to focus on fewer changes, which are possibly initial events of pathogenesis and are clearly relevant for several cell types. These consistent global proteome effects identified putative substrates, but they failed to clarify whether impaired degradation or induced biosynthesis is responsible for the effects, and how the mitochondrial pathology triggers the dysregulation of nuclear and nucleolar factors by retrograde signaling.

### 3.3. Assessment of Transcriptional Induction

**Figure 3:**
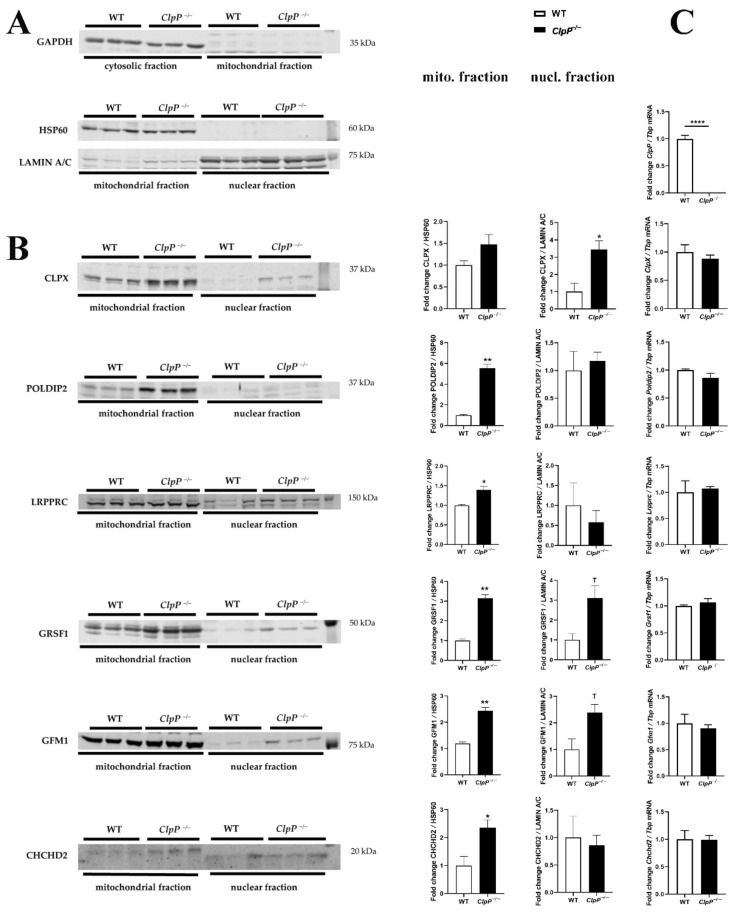
Analyses of redistribution of mitochondrial factors to the nucleus. (**A**) Quantitative immunoblots were used to control subcellular fractionation quality and loading by GAPDH, HSP60, or LAMIN A/C protein abundance. (**B**) Immunoblot validation of protein accumulation in mitochondrial and nuclear fractions (membrane at left margin, bar graph in center). (**C**) Assessment of underlying transcript levels by RT-qPCR (right margin) for specific ClpX interactors in ClpP^−/−^ (*n* = 3) versus WT (*n* = 3) mouse embryonal fibroblasts. * *p* < 0.05, ** *p* < 0.01; **** *p* < 0.0001; T represents 0.05 < *p* < 0.10.

Therefore, we then assessed if these dysregulations are indeed due to altered protein turnover, as a consequence of absent mitochondrial peptidase ClpP, or are due to secondary transcriptional responses that represent compensatory or deleterious downstream events. Therefore, mRNA levels of these crucial factors were quantified by RT-qPCR and shown to be unchanged, with the exception of ClpP mRNA absence (Figure 3). These data indicate that the accumulation of these proteins is not due to nuclear transcriptional upregulation, in agreement with the concept that the protein turnover rate of POLDIP2, LRPPRC, GRSF1, GFM1, and CHCHD2 depends on ClpXP-mediated degradation.

### 3.4. Validation by Immunoblot, with Analysis of Subcellular Distribution

To validate the protein accumulations by quantitative Western blots, and to test whether the ClpP deficiency triggers accumulation of these proteins only within mitochondria or also extra-mitochondrially, subcellular fractionations of MEF cells by differential detergent extraction were performed, as previously established [29]. Fractionation purity was controlled by immunoblots with mitochondrial HSP60, cytosolic GAPDH, and nuclear Lamin A-C, detecting no mitochondrial contamination in the nuclear fraction, and very little nuclear material in the mitochondrial fraction; even the cytoplasmic contamination was minor in these two fractions (Figure 3A).

As expected from the mass spectrometry data, the ClpX-immunoreactive band showed accumulation, but unexpectedly, this effect was much stronger in the nuclear fraction than in the mitochondrial fraction of MEFs (Figure 3B). This observation represents strong evidence that ClpX selectively targets proteins that are associated with DNA or unspliced RNA, so that excess ClpX amounts would find suitable docking sites mainly in the nucleus.

The analysis of the distribution of ClpX interactors POLDIP2 (predicted precursor size 42 kDa) and LRPPRC (predicted precursor size 158 kDa) showed their accumulations to be restricted to mitochondria (Figure 3B).

In contrast, antibodies against GRSF1 (predicted precursor full-length size at 55 kDa) and against GFM1 (predicted precursor size at 83 kDa) showed the expected band to accumulate in mitochondria and the nucleus. The gain-of-function of the mRNA/tRNA/rRNA/lncRNA processing factor GRSF1 and of the tRNA translocation mediator GFM1, both in mitochondria and the nucleus, would probably contribute to mitoribosomal inefficiency. Beyond mitochondrial translation, their excess may also alter nucleolar processing/nuclear export, causing generalized cell stress.

The Parkinson’s disease (PD)-associated UPR^mt^ sensor CHCHD2 (predicted precursor size 16–18 kDa) was increased only in the mitochondrial fraction, and a slightly smaller band in the nucleus did not show dysregulation. CHCHD2 is known to encode two polypeptides, distinguished as PA and PB. The latter is shorter than PA due to the absence of the putative mitochondrial targeting sequence [47]. An antibody of sufficient quality for the sensitive and specific detection of VWA8 in MEFs was not found.

Overall, the accumulations of ClpX and five ClpX interactors were confirmed in mitochondria, and ClpX with two interactors also showed similarly elevated abundance in nuclear fractions.

### 3.5. Conserved ClpX-Dependent Effects in ClpP-Mutant Patient Skin Fibroblasts

Which of these mouse findings can be reproduced in human? Skin primary fibroblast cultures were available to us from two patients with biallelic ClpP variants who had markedly different disease severities (see Section 2). Immunoblots had shown ClpP to be destabilized and absent in patient 58955 [37], making our ClpP mouse mutant a model of this maximal PRLTS3 severity, while the mutation of ClpP in patient 0006 did not affect its abundance and presumably reduces its function only partially, in correlation with the difference in phenotypes above. Thus, the global proteome dysregulations might be expected to be more dramatic in patient 58955 cells than in patient 0006 cells.

**Figure 4:**
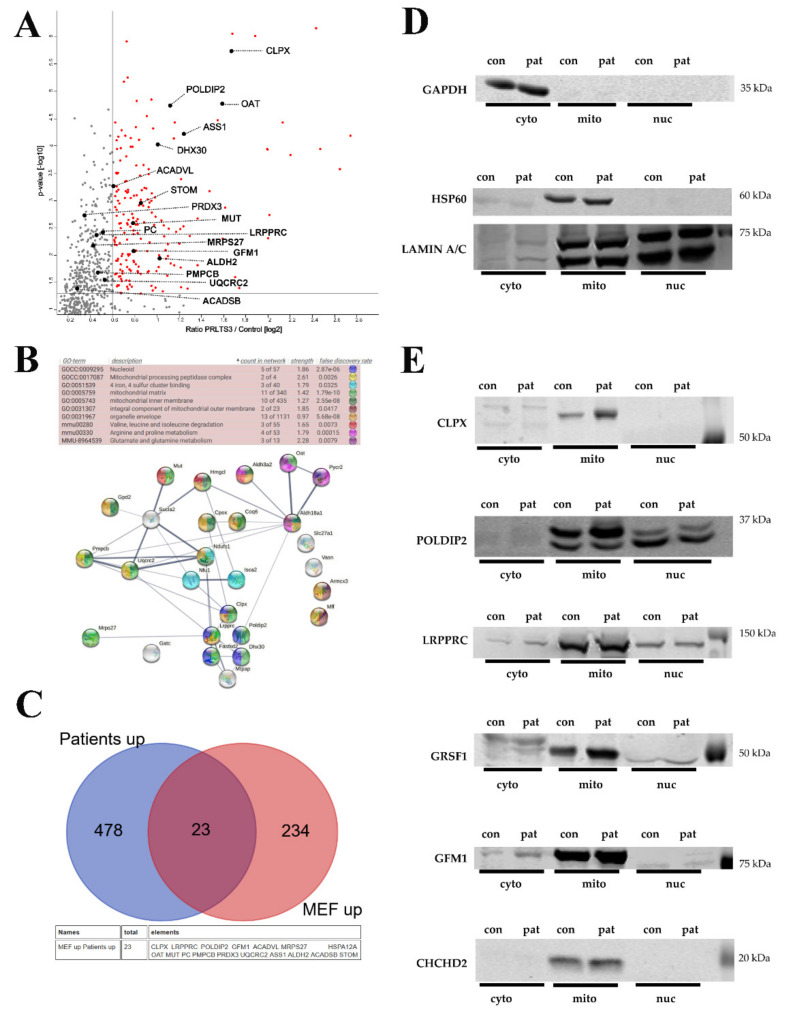
(**A**) Analysis of both PRLTS3 patients’ (in triplicates) versus 7 healthy controls’ skin fibroblast proteome profiles in volcano plots, highlighting the accumulated proteins in mitochondria. The significance threshold at 1.3 on the *Y*-axis corresponds to the *p*-value 0.05, and the *X*-axis shows the traditional cut-off at 1.5-fold effects, although genetic evidence has demonstrated a neurodegenerative process at old age to be triggered by a lower gain-of-function, such as 1.3-fold dosage of alpha-synuclein. (**B**) Analysis of the differences between both patients in a STRING interaction diagram, focused on mitochondrial proteins with significant accumulation in ClpP-mutant patient skin fibroblast global proteome, which showed 1.2-fold stronger change in the severely affected patient 58955 than in the milder patient 0006. (**C**) Comparison of all accumulations with nominal significance in both PRLTS3 fibroblasts that showed consistency with nominal significant accumulations in ClpP-null MEFs, using a Venn diagram. A total of 23 effects were consistent between species, among which all mitochondrial proteins are listed below, employing their gene symbol. (**D**) Analyses of protein abundance in fibroblasts from a human control (red128) and the patient with stronger fold changes (58955), controlling the subcellular fractionation purity and loading by GAPDH (cytosolic marker), HSP60 (mitochondrial), or LAMIN A/C (nuclear) abundance. (**E**) Quantitative immunoblots for CLPX and its protein interactors confirmed their accumulation (e.g., 2.33-fold for ClpX normalized to HSP60) to be restricted to mitochondria.

A completely novel documentation of the global proteome profile of primary skin fibroblasts from these two PRLTS3 patients (average values, each in triplicate culture samples) versus seven healthy controls (Table S6, volcano plot in Figure 4A) failed to detect ClpP levels in every mutant cell triplicate upon mass spectrometry, while ClpP was solidly detected in three out of seven controls. Reanalysis of the samples by immunoblots confirmed ClpP to be almost absent only in the 58955 samples, and present at similar levels in all control samples as well as in 0006 samples. Mass spectrometry found upregulated abundances for the proteins ClpX (average 3.2-fold with *p* = 1.8 × 10^−6^; in the more severe case, higher abundance by 1.7-fold), POLDIP2 (average 2.2-fold with *p* = 0.00002; severe case had 1.5-fold more), LRPPRC (average 1.4-fold with *p* = 0.004; severe case had 1.5-fold more), GFM1 (average 1.7-fold with *p* = 0.008; severe case had 1.7-fold more), GRSF1 (average 5.3-fold with *p* = 0.0002; severe case had 1.1-fold more), and MRPS27 (average 1.1-fold with *p* = 0.02; severe case had 1.4-fold more). Among these factors, ClpP deficiency affected ClpX as well as its interactors POLDIP2 and GRSF1, with a prominent effect size. CHCHD2, VWA8, MRPS9, and MRPS22 proteins were below the detection threshold in this mass spectrometry survey.

Systematic filtering of mitochondrial proteins that were upregulated at least 20% more in the severe case than in the mild case was conducted to define the molecular correlates of disease severity (Figure 4B). Strong enrichment upon STRING statistics was observed for ClpX and other mitochondrial nucleoid-/RNA granule-associated factors (POLDIP2, LRPPRC, FASTKD2, DHX30, MTPAP) [48,49], but not for mitoribosomal proteins. In addition, the individual with the more severe phenotype showed stronger accumulation of mitochondrial protein processing peptidase components, of 4-iron-4-sulfur cluster binding and heme biosynthesis proteins, and of amino acid metabolism enzymes, with significant enrichment upon STRING analysis (Figure 4B; the list of mitochondrial factors with stronger accumulation in case 58955 and their enrichment statistics can be found in Supplementary Table S7). Within amino acid metabolism, the valine/leucine/isoleucine, arginine/proline, and glutamate/glutamine pathway components particularly stood out. Mitoribosomal dysregulation was not prominent or consistent.

Some of these findings seem clearly mediated by ClpX accumulation and can occur without ClpP alteration, in view of a previous report [50] where recombinant ClpX was overexpressed 1.77-fold in mouse C2C12 myoblasts and proteome profiling then detected accumulations of LRPPRC (1.66-fold), POLDIP2 (1.45-fold), GFM1 (1.62-fold), GRSF1 (1.38-fold), FASTKD2 (1.58-fold), and MTPAP (1.41-fold). Thus, the accumulation of factors with a known function at the mitochondrial nucleoid/RNA granule appears to be directly dependent on ClpX excess.

### 3.6. Conserved Potentially ClpX-Independent Effects in ClpP-Mutant Patient Skin Fibroblasts

Testing the consistency between the proteome profiles of both PRLTS3 patient fibroblast cultures (in triplicates) versus ClpP-null MEFs, we also noted significant accumulations of eleven mitochondrial matrix proteins (Figure 4C), which are not known as interactors of ClpX or ClpB: OAT, ASS1, ACADVL, STOM, PRDX3, PC, MUT, ALDH2, PMPCB, UQCRC2, and ACADSB, see Table 2. Importantly, all of them are known for their assembly into homo-multimers or heterodimers [51,52,53,54,55,56,57,58,59,60,61,62]. OAT, PC, ALDH2, PMPCB, and ACADSB also showed significant accumulation in ClpP-null brains (Table S3).

Beyond mass spectrometry, immunoblot analyses in fibroblast protein subcellular fractions from the severely affected PRLTS3 female 58955 (where fold changes in abundance can more easily be visualized in quantitative Western blots) and from the control female red 128 were employed to assess data reproducibility in human (Figure 4D,E). Again, accumulations were apparent for ClpX, POLDIP2, LRPPRC, GRSF1, GFM1 bands, and CHCHD2 in the mitochondrial fraction. A nuclear accumulation of ClpX and its interactors was not observed, even after using a titer of 1:100 of the anti-ClpX antibody that had detected nuclear ClpX in MEF fractions. While it is therefore doubtful if excess ClpX in the nucleus is a relevant event contributing to PRLTS3 pathogenesis, it may modify pathology in the mouse model.

### 3.7. Mass Spectrometry Quantification of Amino Acids in ClpP-Null MEFs

To understand the downstream consequences of the protein accumulations documented above, on the one hand, we assessed the impact of the potentially ClpX-independent accumulation of OAT, ASS1, PC, MUT, and ACADSB on amino acid metabolism, and on the other hand, we assessed the consequences of ClpX interactor excess on the nucleoids. A metabolomics approach by mass spectrometry was taken to quantify the amino acid concentrations in eight ClpP-null versus eight WT MEFs (Table S8, Figure S2), since patient fibroblast lines were not available in sufficient numbers to evaluate mild changes. Although variability was high and future tissue analyses may be needed, the preliminary data showed nominal significance (*p* < 0.05) for increased levels of leucine/isoleucine, contrasting with decreased levels of methionine and histidine. These subtle and selective effects are compatible with the mild phenotype of ClpP-mutant fibroblasts. Overall, literature and our proteome data suggest that specific multimeric complexes are affected (not the bulk of monomeric enzymes) in the mitochondrial matrix of ClpP-mutant cells. However, it has to be taken into account that a 30 kDa cut-off was used in the FASP methodology of our mass spectrometry, so a technological bias might exist here, and further assessment of this observation is necessary.

### 3.8. Nucleoid Misassembly in ClpP-Mutant Patient Fibroblasts

**Figure 5:**
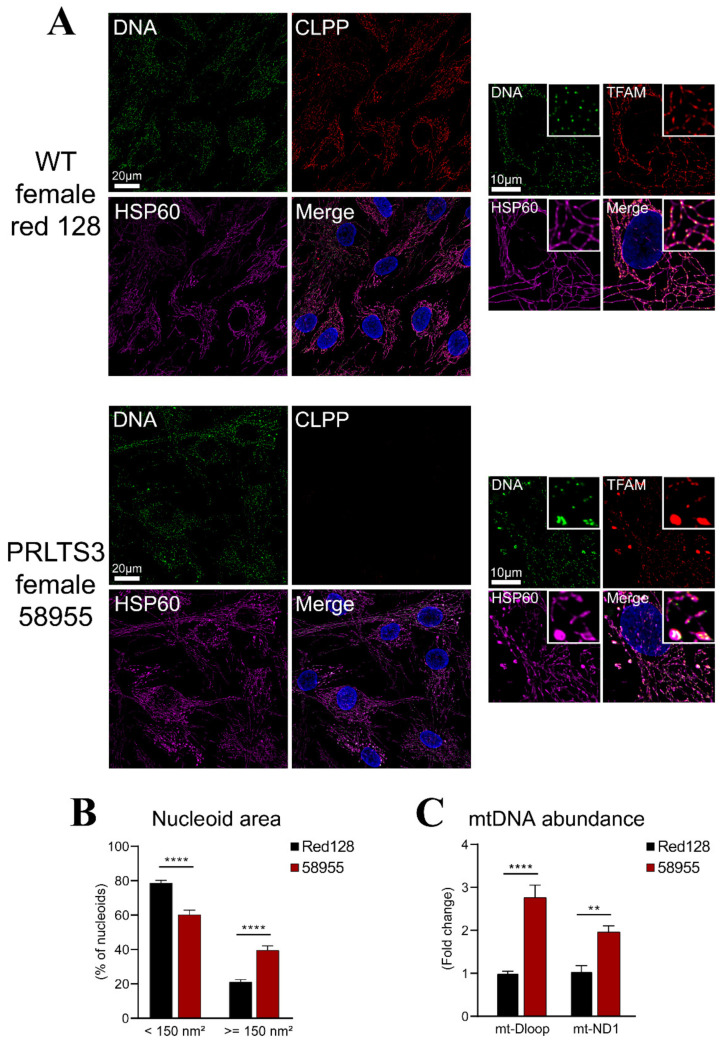
Nucleoid misassembly in PRLTS3 fibroblasts. (**A**) Immunocytochemistry with HSP60 as a mitochondrial marker (purple) and DAPI as a nuclear marker (blue) showed PRLTS3 fibroblasts with a larger TFAM-positive area (red) that contain multiple mitochondrial DNA signals upon deconvolution (green). (**B**) Quantification of nucleoid size showed significant enlargement. (**C**) Copy number quantification by qPCR for two sites within mtDNA also revealed significant increases. ** *p* < 0.01; **** *p* < 0.0001.

Next, we focused on pathology due to the ClpX interactor excess. Our prior work had documented that deficiency of ClpP in mouse cells leads to mtDNA instability and nucleoid enlargement, but whether these features could be recapitulated in Perrault syndrome patients is unknown [17]. Nucleoid staining in human fibroblasts from a control (Red128) and a PRLTS3 patient (CLPP-mutant patient 58955) showed mtDNA stress and packaging alterations in the PRLTS3 patient (Figure 5A), with significant nucleoid enlargement (Figure 5B). Similar to ClpP-null MEFs, PRLTS3 patient cells also showed a significant increase in the mtDNA copy number (Figure 5C), assessed by qPCR with ND1 and DLoop1 mitochondrial DNA primers.

## 4. Discussion

**Figure 6:**
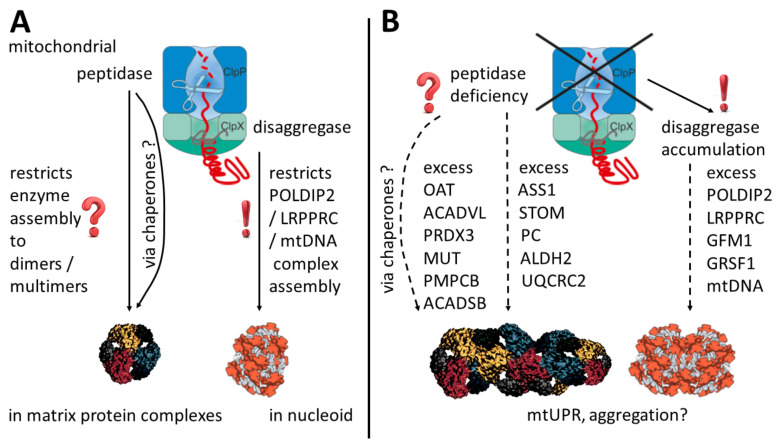
Schematic overview of potential mechanisms in the presence (**A**) and absence (**B**) of ClpP. ClpP inactivating variants act via ClpX accumulation on mitochondrial nucleoid/RNA granule components, triggering excess POLDIP2, LRPPRC, GFM1, GRSF1, and mtDNA abundance, as well as an enlarged nucleoid area, as a clear result of this study. Less consistent across cell types and potentially without ClpX interactions, multimerizing enzymes in the matrix also show increased abundance. In parallel, elevated levels of varying molecular chaperones suggest a mitochondrial unfolded protein response and aggregation tendencies. OAT multimer and nucleoid shapes were adapted from Refs. [63,64].

Our study attempted to define mitochondrial ClpP-mediated degradation substrates in mouse and human. This goal was first approached by consistency criteria, comparing proliferating peripheral cells with glycolytic profiles from two organisms to murine nervous tissue containing postmitotic neurons that have a respiratory profile. To distinguish primary events from secondary consequences, high-quality protein interaction data from the BioGrid database were used to identify the ClpX protein interactors that accumulate upon ClpP inactivity (Figure 1). In a second approach, the fold-changes of accumulation of mitochondrial proteins were correlated with disease severity in two PRLTS3 patients (Figure 4B). Each strategy came to the conclusion that the primary effects of ClpP inactivity involve ClpX interactor proteins at the nucleoid and mitochondrial RNA granule (Figure 6). The fold-changes of accumulation were much stronger for these proteins than what was previously observed for ClpP modulation of mitoribosome components, and, naturally, the observed nucleoid/RNA granule pathology might affect mitoribosomal translation as a downstream consequence. Numerous previous efforts to define ClpP-mediated degradation substrates had been unable to identify consistent results across species and techniques employed, so we wonder to what degree the interactions between ClpX and its target protein/RNA/DNA complexes may be lost during the different co-immunoprecipitation and extraction procedures used.

As a first key finding, ClpP pathogenic variants trigger the protein accumulation of POLDIP2, LRPPRC, and GFM1 with high consistency between mammalian species and cell types. While POLDIP2 functions at the nucleoid and LRPPRC at the mitochondrial RNA granule modulating polyadenylation [41,42,65,66], GFM1 directly acts at mitoribosomes, and its excess was shown to impair translation [44,67,68]. Mitoribosomal dysfunction is considered a crucial site of pathology in Perrault syndrome, and these events occur upstream. In addition, the mouse proteome profiles identified the ClpP-dependent accumulation of GRSF1 as prominent (in ClpP-null MEFs, 4.6-fold and highly significant; in ClpP-null brain pellets, 2.1-fold with actual significance; in ClpP-null brain supernatants, 1.8-fold with a statistical trend *p* = 0.06; in human fibroblasts, it was detected only in two out of seven controls and was therefore ignored by the filtering algorithm, but imputation revealed a >5-fold highly significant accumulation). GRSF1 acts with RNase P to process mitochondrial polycistronic precursor RNA into tRNAs/mRNAs/rRNAs and lncRNAs [43,69]. GRSF1 associates with Twinkle in the mitochondrial RNA granule [5] and controls the translation of mRNAs with G-repeats in front of the start codon [70,71]. Thus, GRSF1 connects ClpXP with Twinkle and tRNA maturation in mitochondrial RNA granules. Our crucial novel insight of ClpP-dependent GFM1 and GRSF1 abundance therefore suggests a common pathway for Perrault syndrome pathogenesis, since the genetic causes of autosomal recessive Perrault syndrome include variants in the mitochondrial tRNA amino acid synthetases HARS2 and LARS2, the mitochondrial tRNA processing enzyme PRORP, the mitochondrial DNA/RNA helicase/primase TWNK, and the peptidase ClpP. It is noteworthy that none of the Perrault syndrome disease proteins were dysregulated, except HSD17B4 that showed a significant upregulation only in patient fibroblasts, meaning the role of ClpP cannot be explained by abnormal degradation of a Perrault syndrome disease protein, by this dataset.

A second key finding is the relocation of excess amounts of ClpX and its interactors such as GFM1 and GRSF1 outside mitochondria. This might represent an additional mechanism of how retrograde signals about mitochondrial needs and pathology reach the nucleus, in the context of UPR^mt^ but independent of transcriptional regulation, influencing the abundance of nuclear/nucleolar proteins and affecting the whole cell. This ClpX redistribution was observed only in mouse and was not demonstrable in patient fibroblasts. As a similar difference between mouse and human, it is interesting to note that the ClpP-null MEFs showed a massive induction of the innate immune defense against toxic DNA/RNA, while the patient fibroblasts did not show such immunostimulation. GRSF1 has a physiological presence in the nucleus, as with other members of the hnRNP F/H family [72], but its function there is not clear. GRSF1 abundance is physiologically controlled by DAZL [73], the master translational regulator of spermatogenesis [74,75]. It is known that a G4-repeat DNA structure resolvase named RHAU is essential for spermatogonia differentiation via c-Kit [76]. Furthermore, GRSF1 targets the G-repeat-containing mRNA of GPx4 [77], a crucial factor for protamine-mediated chromatin condensation during spermatogenesis [78]. Thus, it is conceivable that dysregulated GRSF1 abundance interferes with germ cell differentiation, meiosis, and sperm capacitation in PRLTS3. Primary ovarian insufficiency is also observed upon mutation of the mitochondrial tRNA synthetases HARS2, LARS2, and PRORP, meaning that, apparently, other mitochondrial tRNA processing alterations may also influence meiotic events in the nucleus.

The weakest effect of ClpP deletion on a ClpX interactor in the above experiments was observed for the PD-associated UPR^mt^ sensor CHCHD2 [25,45], in agreement with previous observations that ClpP-null mice do not show widespread strong UPR^mt^ [79] or the typical distribution of PD pathology [2,80].

As a third key finding, ClpP deletion across species and cell types consistently triggers the accumulation of homo/hetero-multimerizing enzymes in the mitochondrial matrix such as OAT, ASS1, ACADVL, STOM, PRDX3, PC, MUT, ALDH2, PMPCB, UQCRC2, and ACADSB. ClpP-dependent OAT accumulation stands out and was already proposed, together with POLDIP2, as a putative ClpP substrate in a previous mouse fibroblast proteome study [12], although it was never found to interact with ClpX. The putative ClpP substrate PMPCB forms heterodimers with PMPCA and has a key role in mitochondrial precursor protein cleavage/folding as well as presumably UPR^mt^, in a very similar manner to its homologous cleavage factor UQCRC2 that forms heterodimers with UQCRC1 and is crucial for respiratory complex III assembly. The observation of normal levels for valine, arginine/proline, glutamate/glutamine, and, in particular, ornithine in our ClpP-null MEFs suggests normal enzymatic activity remains for OAT even though it is accumulated and although the OAT hexamer might be misassembled. Additionally, a previous study of *Podospora anserina* ClpP deletion mutants also observed increased leucine/isoleucine, as opposed to decreased histidine levels [81]. The previously reported metabolome data in PRLTS3 patient 58955, where urinary excretion contained excess amounts of fumarate and oxo-glutarate [37], suggest that carbon flux in the TCA cycle occurs at high levels, possibly at the expense of specific amino acids as well as glucose. Abnormal assembly of UQCRC2 in mitochondrial complex III could easily explain why its respiratory activity was mildly low in a muscle biopsy of PRLTS3 patient 58955 [37]. High-throughput phenotype screening of the ClpP-null mouse organism previously revealed decreases in circulating glucose and insulin levels versus increases in circulating cholesterol and bilirubin levels (https://www.mousephenotype.org/data/genes/MGI:1858213#phenotypesTab, accessed on 27 October 2021). Additionally, studies of the metabolome in the ClpP-deficient microorganism *Bacillus subtilis* showed elevated levels for most glycolytic metabolites and TCA intermediates [13,82,83,84].

Although the accumulation of MUT was mild in our mass spectrometry and immunoblot data, it correlates best with the metabolome findings of elevated methylmalonyl-CoA and isoleucine levels in ClpP-mutant fibroblasts. MUT acts on methylmalonyl-CoA that is derived from degraded branched-chain amino acids such as isoleucine, isomerizing it to succinyl-CoA as a substrate for the TCA cycle. Given that MUT has to assemble into homodimers with GTPase-assisted binding to vitamin B12 [85], its optimal activity may not be achieved upon chaperone/disaggregase excess, leading to accumulations of dysfunctional MUT, its substrate methylmalonyl-CoA, and its precursor isoleucine. The reduced levels of methionine as a start codon for protein synthesis might mirror inefficient ribosomal translation. Alternatively, the reduction in methionine together with histidine might point to altered glucogenic metabolism. However, we want to caution against over-interpretation of our metabolomics findings, since 3 alterations with nominal significance among the 54 metabolites investigated might simply be explained away as multiple testing artifacts, or a false positive result.

The accumulation of these proteins that have to multimerize after mitochondrial import could represent a secondary consequence of the UPR^mt^ stress in fibroblasts that predominantly use glycolysis, where altered abundance of varying molecular chaperones and altered pH can conspire to impair the maturation of functional multimer complexes. However, in the light of our search for ClpP substrates that accumulate in PRLTS3 cells, we might also assess these factors as potential primary targets of ClpP dysfunction, possibly independent of ClpX. Thus, these proteins might shed light on ClpP’s basic function. It is important to know that ClpP, by itself, has the capacity to digest small peptides, preferentially with hydrophobic/aromatic residues, in a chymotrypsin-like manner [13,83] and it is therefore classified as a peptidase, until its association with ClpX enables it to degrade complete proteins as a protease. Thus, it is conceivable that the specificity pockets of ClpP heptamers target hydrophobic peptides in between target multimeric proteins to cleave their interaction domains and regulate their activity, as a rapid stress response inside mitochondria long before the cell nucleus can induce alternative allelic splice isoforms of mitochondrial factors that may compensate stress. Such a phenomenon has been reported for the nucleoid-interacting protein Twinkle, whose mutations can also trigger Perrault syndrome, and which was unfortunately not detected in our mass spectrometry dataset with current methods. The hexameric structure and nucleoid associations of Twinkle depend on its C-terminal sequences, and an alternatively spliced isoform named Twinky was observed to exist as a monomer that is diffusely distributed throughout the mitochondrial matrix [84]. Clearly, the role of ClpP for Perrault syndrome might become more understandable if its dysfunction controls the abundance/multimerization/target interaction/activity of the PRLTS disease protein Twinkle at the nucleoid. Thus, we postulate that a hypothetical role of ClpP peptidase for target multimerization is compatible with current knowledge and relevant enough to be investigated deeply in future targeted experiments. Altogether, our proteome profiles and BioGrid data are compatible with a scenario where ClpP, by itself, acts, e.g., on the OAT homohexamer that is stably associated with pyridoxal phosphate in the mitochondrial matrix, and ClpB assists the disaggregation of mitoribosomes, while ClpX mediates the targeting of mitochondrial nucleoids together with adjacent RNA granules. ClpX might select substrates for ClpP-mediated cleavage during the assembly and disassembly of mitochondrial DNA/rRNA/mRNA/tRNA with proteins in these complexes.

As a fourth key finding, human cells from ClpP-mutant PRLTS3 patients show an enlarged nucleoid area and thus confirm the significant role of ClpP in the maintenance of mtDNA/protein complex assembly and distribution, which was previously observed in mouse cells with ClpP deficiency. Here, it is interesting to note that ClpX was previously reported to modulate the genome distribution in mitochondria, and to enhance the DNA-binding activity of TFAM as a requirement for maintaining a normal mitochondrial nucleoid structure [86]. The phenotype observed in PRLTS3 patient cells indicates a potential role for mtDNA stress in the pathogenesis of Perrault syndrome, a scenario that has not been explored before. These findings indicate a conserved role for ClpP in mtDNA homeostasis that might have implications for downstream mitochondrial transcription and translation, and for downstream activation of innate immunity via cGAS.

Considering all data and the literature, we speculate that the selective accumulation of mtDNA copies in nucleoids and of multimerizing enzymes in the matrix in ClpP-mutant cells may suggest that ClpP acts to restrict unwanted further self-assembly, once the appropriate degree of multimerization or the appropriate number of supercoiled DNA rings per nucleoid is reached. According to this notion, ClpP function would limit multimerization and would counteract aggregation and fibril formation in the mitochondrial matrix, rather than having a responsibility for the degradation of bulk proteins, as with the mitochondrial matrix protease LonP1.

Overall, the present data concur with previous evidence that the PRLTS3 pathology is dominated by improper handling of nucleic acids by proteins, leading to nucleoid pathology in mouse and human, as shown here, as well as distorted responses of cytosolic immune sensors to toxic nucleic acids, at least in mouse cells, as reported before [17,29]. In comparison, the decrease in metabolites is minor and does not seem to explain the substantial growth deficit of ClpP-deficient organisms.

## 5. Conclusions

Overall, our analysis of accumulated mitochondrial factors in ClpP mutants, which are consistent across species and cell types, defined several putative substrates of ClpP-mediated degradation. Among them, POLDIP2, LRPPRC, and GFM1 interact with ClpX disaggregase and are therefore particularly credible as primary targets of ClpXP function; their roles for mitochondrial nucleoids and RNA granules explain how nucleoids become misassembled in PRLTS3, as the most important functional impact revealed by our study. The excessive amounts of ClpX and its interactors such as GFM1 and GRSF1 relocalize to the nucleus in ClpP-null mouse fibroblasts, possibly redistributed there by the large amount of protein/DNA/RNA complexes as docking stations. In contrast, the fibroblast-selective accumulation of multimerizing, often vitamin-associated factors such as OAT, MUT, PMPCB, UQCRC2, PC, ACADVL, PRDX3, ASS1, ALDH2, ACADSB, and STOM in ClpP-null mice may be an indirect downstream feature due to the dysregulation of molecular chaperones under conditions of UPR^mt^, or it might potentially be a direct effect independent of ClpX, but it clearly has only a minor impact on metabolism under unstressed conditions.

## Figures and Tables

**Table 1:** Mitochondrial proteins (UniProt-IDs) with significant downregulation (upper part) and upregulation (lower part) in ClpP-null MEFs versus WT (3 versus 3). Factors with special relevance for this manuscript are highlighted in bold letters. Previously reported candidate ClpP cleavage substrates in mouse are identified at the right margin with citations of the relevant PubMed-ID number.

Downregulations				
Majority Protein IDs	Gene Names	*p*-Value	Fold Change with Imputation	
Q8BWT1;Q3UKH3	Acaa2	0.0009	−1.23	
Q8JZN5	Acad9	0.0024	−1.26	
F6 × 5P5;Q6PE15	Abhd10	0.0029	−1.98	
Q64429;Q80V82;Q3UTK2;Q9CUA1;Q8BRY0	Cyp1b1	0.0047	−6.59	
P32020	Scp2	0.0048	−1.31	
Q3UGW4;O88696;Q8CF81	**Clpp**	0.0065	−14.78	
Q3V471;Q8C253;P16110	Lgals3	0.0077	−1.18	
O08528;E9Q5B5	Hk2	0.0098	−1.34	
Q923F9;E9QPX3;Q9CXZ1;Q9CTT4	Ndufs4	0.0119	−1.26	
Q9CRB9;S4R238;Q9D9P1;D3Z0L4;Q91VG6	Chchd3	0.0161	−1.14	
Q91YP2;Q3UUI1;Q3TB65	Nln	0.0186	−1.30	
Q9CQ54;Q9D846	Ndufc2	0.0194	−1.28	
P48771	Cox7a2	0.0195	−2.71	
Q9Z2I8;C6EQH3	Suclg2	0.0206	−1.44	
Q9CQB5;D3Z3X4	Cisd2	0.0211	−1.23	
Q6GQU1;Q3UPC4;Q3TJE3;G3UVV4;P17710;Q3UE51;Q3TTB4	Hk1	0.0218	−1.11	
X2EXD0;Q920L1	Fads1	0.0226	−1.44	
A0A0R4J0G0;Q8BH04;Q8R3X7;Q3UGF0	Pck2	0.0304	−1.20	
Q9CS68;Q9WV84	Nme4	0.032	−1.55	
Q8BH95;F6T930	Echs1	0.0352	−1.23	
Q9CYT3;Q99N15;A2AFQ2;O08756	Hsd17b10	0.0374	−1.21	
A2AP32;A2AP31;Q3UIU2	Ndufb6	0.042	−1.42	
Q9WTP7;Q9D8W6	Ak3	0.0423	−1.13	
Q9D6U8	Fam162a	0.0427	−1.36	
Q9D0K2;Q3UK61;Q3U9P7;Q3UJQ9;Q9CRF4	Oxct1	0.0475	−1.20	
Q8K009;D3Z6B9	Aldh1l2	0.0496	−1.38	
Upregulations				
**Majority Protein IDs**	**Gene Names**	***p*-Value**	**Fold Change with Imputation**	**Previous Mammalian Substrates PMID**
P38647	Hspa9	2E-07	1.41	32467259
E9Q179;Q8C5Q4;Q8C298	**Grsf1**	3E-05	4.64	
Q60597;Z4YJV4	Ogdh	9E-05	1.16	
O35459;F7B227	Ech1	1E-04	2.35	
Q8CCC0;Q3TZ21;Q9DCJ7	Aurkaip1	0.0003	3.20	
Q8K0D5	Gfm1	0.0003	2.62	
F6SQH7;Q91VA6	**Poldip2**	0.0003	2.08	32467259
Q3UJ34;Q3UEJ7;P16460;J3QNG0	Ass1	0.0005	1.80	
A0A0M3HEP3;Q8CCZ4;D3Z6I3;Q99M04	Lias	0.0008	4.07	
Q8C6I2	Sdhaf2	0.0009	7.70	
Q3TG75;P29758;Q3UKT3;Q3UJK5	**Oat**	0.0012	2.09	32467259
Q9DB77	**Uqcrc2**	0.0013	1.48	
Q3TJA9;Q99M87	Dnaja3	0.0016	2.33	
F7ASG0;Q9CWV0	Malsu1	0.0017	4.73	
Q8CC88	Vwa8	0.002	1.74	
A0A0A0MQD1;Q8K1H1	Tdrd7	0.0023	1.51	
Q8BK72;Q80ZI4	Mrps27	0.0024	1.72	
Q6PB66	**Lrpprc**	0.0027	1.33	
Q3TIC8;Q9CZ13;Q3THM1;Q8K2S8	**Uqcrc1**	0.0033	1.65	32467259
Q7TMY2;Q9DBL1;E9Q5L3;Q3V2R9	Acadsb	0.0033	1.33	
Q3TI14;Q99LZ4;Q8VE22;F7ARZ1;A7M7Q8;Q3THH3	Mrps23	0.0034	1.67	
Q8K2B3	Sdha	0.0036	1.36	
P20108	**Prdx3**	0.0039	1.29	
Q3UCB5;Q3UC13;O55028;Q8C6H9;D3Z7R0;Q99KP1	Bckdk	0.0053	3.98	
Q61102	Abcb7	0.0066	1.62	
Q99N96;Q9D3F3;Q9CUL8	Mrpl1	0.0066	1.60	
Q922Z3;Q3UPJ8;Q9CQN1;Q922R9;Q3TK29;Q3TSG8	Trap1	0.0074	1.62	
Q3URE1	Acsf3	0.0074	1.25	
Q9CQE3;D3Z198;D3YWF8	Mrps17	0.0079	1.75	
Q99LP6	Grpel1	0.0081	1.67	
Q9D7N3;Q3UMV5;Q9ER89;Q5XJX8	Mrps9	0.0093	1.58	
Q810B1;Q6P060;Q5RL20;Q99N89	Mrpl43	0.0097	2.64	
P18155;Q3U8E7	Mthfd2	0.0131	2.80	
P16332;Q8VED0	**Mut**	0.0135	1.37	
Q3UTB8	Grpel1	0.0136	3.26	
Q544B1;Q3UJW1;Q3U9J7;P47738;A0A0G2JEU1;Q3U6I3;Q3TVM2	Aldh2	0.014	1.53	
Q3TPD9;Q3U2X5;Q64133;Q3UJ53	Maoa	0.0164	1.17	
G5E8R3;Q3T9S7;E9QPD7;Q3TCQ3;Q05920;Q3UFS6;Q62043;Q8BP54	**Pcx;Pc**	0.0165	1.27	
Q80X85	Mrps7	0.0173	1.26	
Q3U9C4;Q3UKG1;Q9CY73	Mrpl44	0.0185	1.41	
D3Z5B1;Q8CEW7;B2RPU8;Q9D1L0	Zbed5;**Chchd2**	0.0193	1.47	
Q9JK42	Pdk2	0.0212	1.72	
Q9CQ06	Mrpl24	0.0216	2.08	
Q3TFD0;Q9CZN7;Q99K87	Shmt2	0.0217	1.21	
E9Q0A7;Q4KMM3	Oxr1	0.022	1.37	
Q9CXW2	Mrps22	0.0236	1.87	
A0A0N4SUH8;Q9QZ23	Nfu1	0.025	1.58	
E0CX98;Q9D2C7;E0CZE8;E0CXR0	Tmbim6	0.0251	1.71	
Q6P8N8;Q9JHS4	**Clpx**	0.0296	1.77	
Q545U2;Q9Z0V8;D3Z1Z0	Timm17a	0.0296	1.29	
Q9CQ40;Q8BTB8	Mrpl49	0.03	1.25	
Q9JKF7;Q8CCX9	Mrpl39	0.0305	3.35	
Q9CXT8;Q3TET5	**Pmpcb**	0.0313	1.42	
P50544;B1AR28;Q3UJR6	**Acadvl**	0.0313	1.23	
B1B1D8;Q9D773	Mrpl2	0.0322	1.71	
Q924T2;Q8BQ99	Mrps2	0.0327	1.65	
Q9CQZ5	Ndufa6	0.0341	1.20	
Q8BTQ6;Q9CQL5	Mrpl18	0.035	5.01	
Q8BK51;A2AIW9;Q3UDE0;Q3TY06;Q3TTM6;Q9DC61	Pmpca	0.035	1.33	
Q9D6Z0	Alkbh7	0.0351	5.84	
Q545A2;P51881	Slc25a5	0.0355	1.22	
Q8CG72	Adprhl2	0.0422	1.09	
Q9DCB8	Isca2	0.0435	2.37	
Q4FJR4;Q3V250;Q922H2	Pdk3	0.0445	1.96	
Q7TMQ1;P23242	Gja1	0.0451	1.59	

**Table 2:** Significant accumulations of the above eleven mitochondrial matrix proteins were consistent between both PRLTS3 fibroblasts and ClpP-null MEFs, and partially consistent with ClpP-null brains (highlighted by gray background), although they are not known as interactors of ClpX or ClpB. All of them show a multimerized quaternary structure, and they partly associate with cofactors.

Gene Symbol	Function	Pathway	Cofactor	Quaternary Structure	Reference	PRLTS3 Fib. Accumulation *p*-Value	PRLTS3 Fib. Accumulation Fold Change
OAT	ornithine amino-transferase	amino acid metabolism/urea cycle	pyridoxal- phosphate	homo- tetramer	55	0.00002	3.0
ASS1	arginino-succinate synthase	urea cycle	-	homo- tetramer	61	0.00006	2.4
ACADVL	very-long-chain acyl-CoA dehydrogenase	fatty acid degradation	flavin-adenine- dinucleotide (FAD)	homo-dimer	59	0.0005	1.5
STOM	ion channel activator	innate immune defence	-	homo-dimer or homo- oligomer	82	0.001	1.8
PRDX3	antioxidant	selenium & senescence	-	homo-dimer & dodecamer	60	0.002	1.3
PC	pyruvate carboxylase	amino-fatty acid/glucose metabolism	biotin	homo- tetramer	58	0.003	1.7
MUT	methyl-malonyl-CoA mutase	carbon metabolism	adenosyl- cobalamin	homo-dimer	53, 54	0.004	1.4
ALDH2	aldehyde dehydrogenase	alcohol- oxidation, CoA biosynthesis	-	homo- tetramer	62	0.01	2.0
PMPCB	mitochondrial targeting sequence cleavage	proteostasis	-	hetero-dimer with PMPCA	56	0.02	1.4
UQCRC2	cleavage of complex III components	respiratory chain assembly	-	hetero-dimer with UQCRC1	57	0.03	1.4
ACADSB	short/branched chain specific acyl-CoA dehydrogenase	amino acid metabolism	flavin-adenine- dinucleotide (FAD)	homo- tetramer	81	0.04	1.2

## Data Availability

Proteome profiles of MEFs and human fibroblasts were deposited to the ProteomeXchange Consortium via the PRIDE partner repository with the dataset identifiers PXD023677 (MEFs) and PXD029418 (human fibroblasts) (Project Webpage: http://www.ebi.ac.uk/pride/archive/projects/PXD023677, accessed on 29 October 2021).

## Supplementary Materials

The following are available online at https://www.mdpi.com/article/10.3390/cells10123354/s1, Figure S1. Factors with significant (*p* < 0.03) accumulation in the global proteome profile (valid values) of ClpP-null MEFs. Figure S2. Analyses of protein abundance for two multimerizing proteins in MEFs (panel A, *n* = 3) and in human control and patient fibroblasts (panel B, #58955, *n* = 1). Table S1. Global proteome data of WT and ClpP-null MEF cells (*n* = 3). Table S2. Pathway enrichment analyses for non-imputed dysregulations (*p* < = 0.03) in global proteome profile of ClpP-null versus WT MEF cells, according to the STRING webserver. Table S3. Global proteome data of ClpP-null versus WT brain tissue (*n* = 6 versus 6), comparing RIPA-soluble supernatant and the resulting pellet dissolved in SDS. Table S4. Pathway enrichment analyses in the global proteome profile of ClpP-null versus WT brain tissue, STRING webserver output. Table S5. BioGRID output of protein interactors for human ClpP, ClpX, and ClpB. Table S6. Global proteome profile of primary skin fibroblasts (control *n* = 7, PRLTS3 *n* = 2 lines in biological triplicates). Table S7. Mitochondrial factors with significant accumulation in ClpP-mutant patient fibroblasts, which was at least 20% stronger in the severe PRLTS3 patient 58955 than in the milder patient 0006. Table S8. Metabolome data of ClpP-null versus WT MEFs (*n* = 8).

## Abbreviations

ACADSB	Acyl-CoA Dehydrogenase, Short/Branched Chain Specific
ACADVL	Acyl-CoA Dehydrogenase, Very-Long Chain
ALDH2	Aldehyde Dehydrogenase, Mitochondrial Isoform
ANOVA	analysis of variance
ASS1	Argininosuccinate synthase 1
BCA	bicinchoninic acid assay
cGAS	Cyclic GMP-AMP Synthase
CHCHD2	Coiled-Coil-Helix-Coiled-Coil-Helix Domain Containing 2
c-kit	tyrosine-protein kinase KIT
ClpB	Caseinolytic Mitochondrial Matrix Peptidase Chaperone Subunit B
ClpP	Caseinolytic Mitochondrial Matrix Peptidase Proteolytic Subunit
ClpX	Caseinolytic Mitochondrial Matrix Peptidase Chaperone Subunit X
DAZL	Deleted In Azoospermia Like
DMEM	Dulbecco’s modified Eagle medium
Dnaja3	DnaJ Heat Shock Protein Family (Hsp40) Member A3
EDTA	Ethylenediaminetetraacetic acid
Eral1	Era Like 12S Mitochondrial RRNA Chaperone 1
ESI	electrospray ionization
FBS	fetal bovine serum
GAPDH	Glyceraldehyde-3-Phosphate Dehydrogenase
GFM1	G Elongation Factor Mitochondrial 1
GPx4	Glutathione Peroxidase 4
GRSF1	G-Rich RNA Sequence Binding Factor 1
HARS2	Histidyl-TRNA Synthetase 2, Mitochondrial
hnRNP	Heterogeneous Nuclear Ribonucleoprotein
Hsd17b4	Hydroxysteroid 17-Beta Dehydrogenase 4
HSP60	Heat Shock Protein Family D (Hsp60) Member 1
LARS2	Leucyl-TRNA Synthetase 2, Mitochondrial
lncRNA	long non-coding RNAs
LRPPRC	Leucine Rich Pentatricopeptide Repeat Containing
MEFs	mouse embryonic fibroblasts
MRM	multiple reaction mode
mRNA	messenger RNA
MRPS22	Mitochondrial Ribosomal Protein S22
MRPS27	Mitochondrial Ribosomal Protein S27
MRPS9	Mitochondrial Ribosomal Protein S9
mtDNA	mitochondrial DNA
MUTn	Methylmalonyl-CoA Mutasetotal sample number
OAT	Ornithine Aminotransferase
OXR1	Oxidation Resistance 1
PC	Pyruvate Carboxylase
PCR	polymerase chain reaction
PD	Parkinson’s disease
PMPCA	Peptidase, Mitochondrial Processing Subunit Alpha
PMPCB	Peptidase, Mitochondrial Processing Subunit Beta
POLDIP2	DNA Polymerase Delta Interacting Protein 2
PRORP	Protein Only RNase P Catalytic Subunit
PRLTS	Perrault syndrome
RHAU	RNA Helicase associated with AU-rich element
RIPA	Radioimmunoprecipitation assay buffer
RNA	ribonucleic acid
rRNA	ribosomal RNA
SDS	sodium dodecyl sulfate
SEM	standard error of the mean
STING	Stimulator Of Interferon Genes
STOM	stomatin
STRING	Search Tool for Recurring Instances of Neighboring Genes
TCA cycle	tricarboxylic acid cycle
Tbp	TATA-Box Binding Protein
tRNA	transfer RNA
Twnk	Twinkle
UPRmt	unfolded protein response, mitochondrial
UQCRC2	Ubiquinol-Cytochrome C Reductase Core Protein 2
UQCUC1	Ubiquinol-Cytochrome C Reductase Core Protein 1
VWA8	Von Willebrand Factor A Domain Containing 8
WT	wild type
ZFE	central animal facility
