# Supplementary material for: Inactivity of Peptidase ClpP Causes Primary Accumulation of Mitochondrial Disaggregase ClpX with Its Interacting Nucleoid Proteins, and of mtDNA
Source: Cells. 2021 Nov 29;10(12):3354. doi: 10.3390/cells10123354 (PMC8699119; doi:10.3390/cells10123354)
Supplement: Supplementary file 1 [file cells-10-03354-s001.zip › cells-1469176-supplementary resubmitted-final/Supplementary figures.pdf]

MMU-392499	Metabolism of proteins	16 of 401	0.72	4.10e-06
KW-0496	Mitochondrion	44 of 1103	0.72	1.99e-17
KW-0143	Chaperone	7 of 196	0.67	0.0108
mmu00980	Metabolism of xenobiotics by cytochrome P450	4 of 65	0.9	0.0346
KW-0411	Iron-sulfur	4 of 62	0.93	0.0161
PF10436	Mitochondrial branched-chain alpha-ketoacid dehydrogenas...	2 of 5	1.72	0.0336
PF01186	Lysyl oxidase	2 of 5	1.72	0.0336
PF00890	FAD binding domain	3 of 19	1.31	0.0297
IPR015422	Pyridoxal phosphate-dependent transferase domain 1	3 of 36	1.04	0.0411
IPR036465	von Willebrand factor A-like domain superfamily	5 of 100	0.81	0.0359

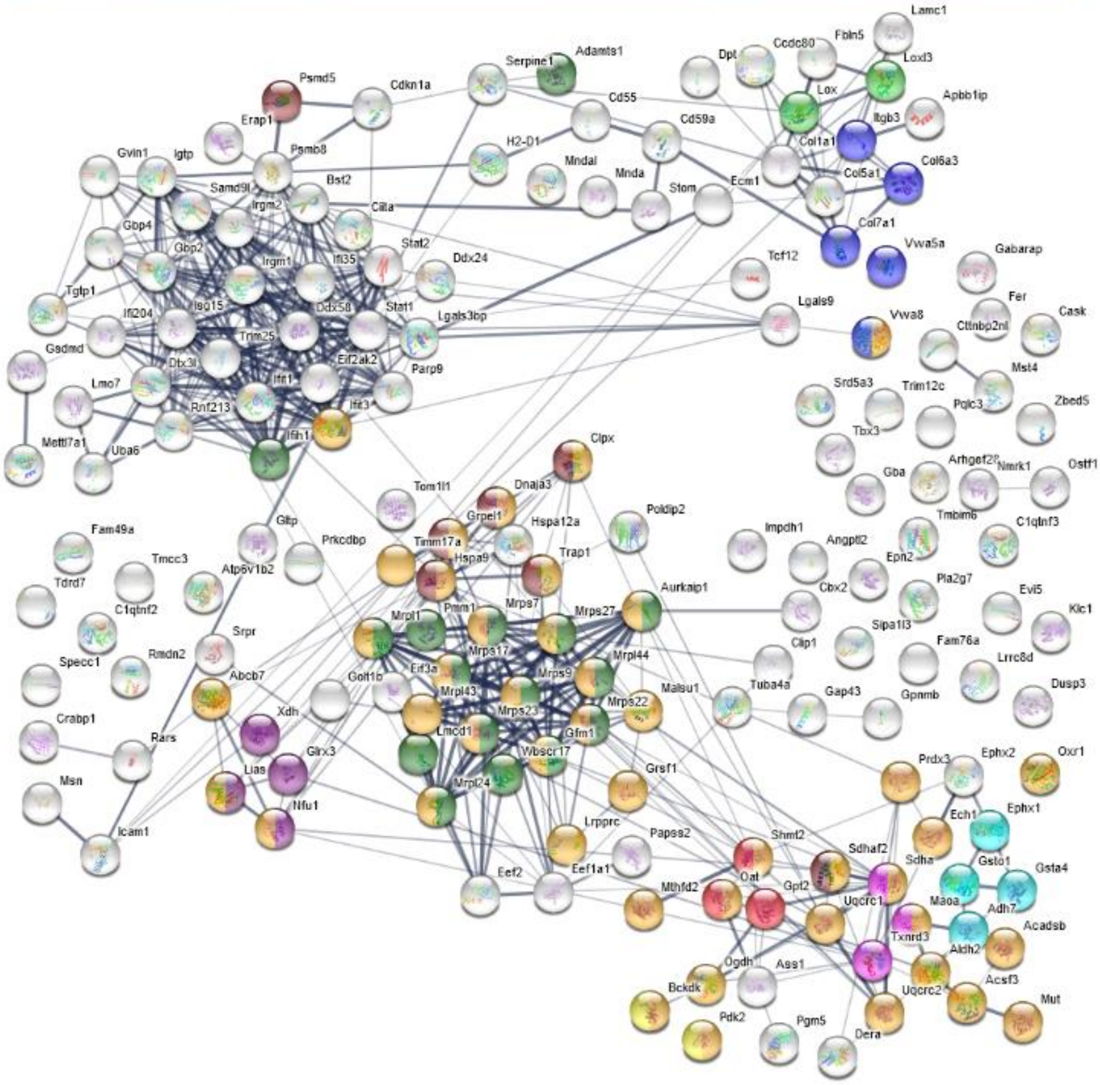

Supplementary Figure S1. Factors with significant (p<0.03) accumulation in the global proteome profile (valid values) of ClpP-null MEFs. The diagram was generated on the STRING web-platform to illustrate their interactions in functional networks. Prominently affected pathways (color code and significance are shown in the upper part of the figure) include the Metabolism of proteins (e.g. mitoribosomal subunits), Chaperones (usually associated with ClpX), Mitochondrion (including also the Iron-sulfur-cluster biogenesis such as LIAS or NFU1, and the Branched-chain amino acid / alpha keto-acid dehydrogenases such as BCKDK, as well as FAD-binding domain proteins such as SDHA, and Pyridoxal phosphate-dependent transferase domain 1 containing factors such as OAT, or several von Willebrand factor A-like domain superfamily members such as mitochondrial VWA8 with its nuclear counterpart VWA5A), and the Cytochrome P450-dependent metabolism of xenobiotics (which depends on mitochondrially synthesized heme).

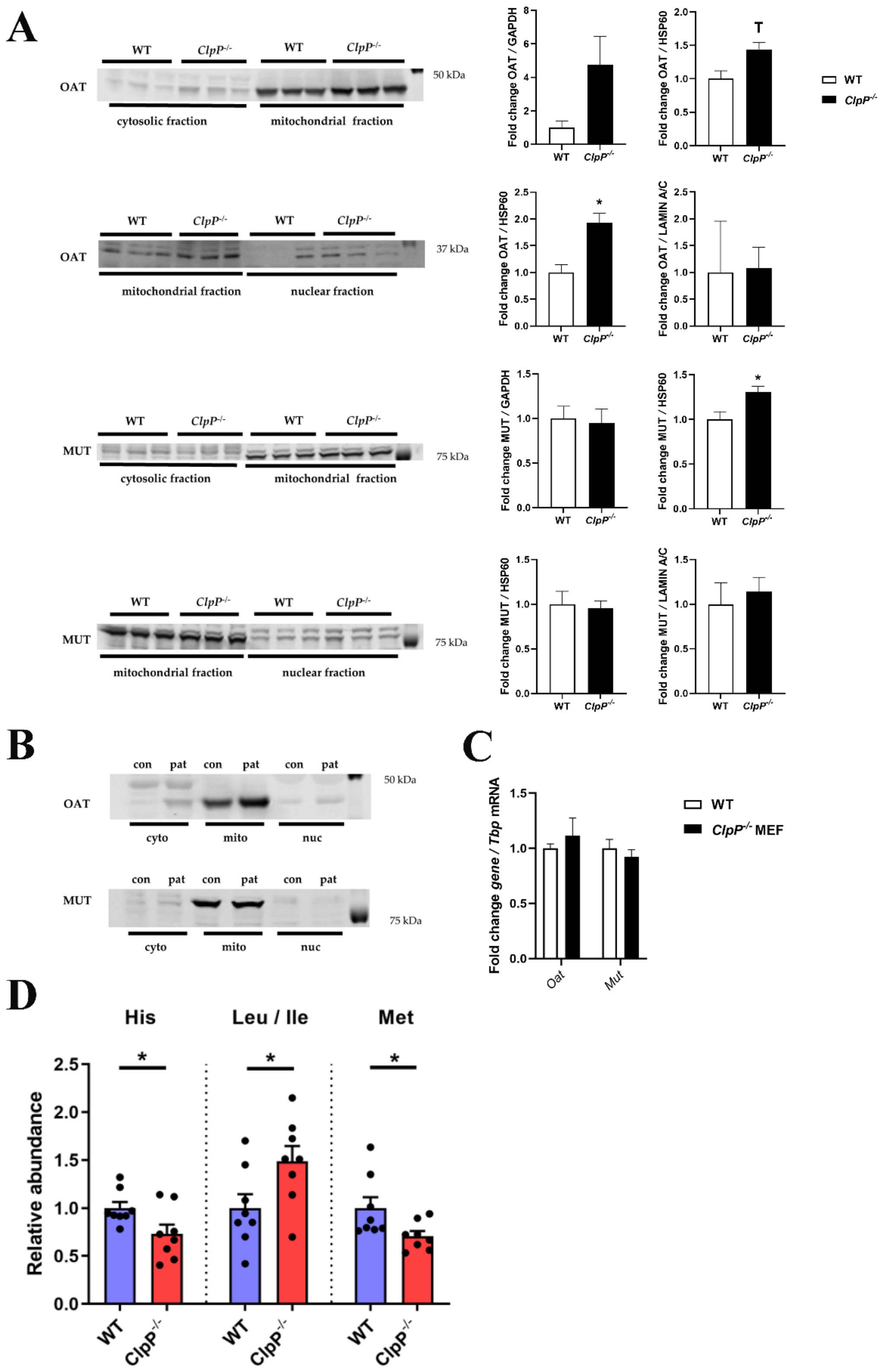

Supplementary Figure S2. Analyses of protein abundance for two multimerizing proteins in MEF (panel **A**, n=3) and in human control and patient fibroblast (panel **B**, #58955, n=1). Subcellular fractionation quality and loading were controlled by GAPDH, HSP60, or LAMIN A/C abundance. Panel **C**: mRNA quantification via RT-qPCR. Expression data were normalized against *Tbp* mRNA levels. Panel **D**: Quantification of 53 metabolites in MEF via LC-tandem mass spectrometry revealed 3

dysregulations with nominal significance. Absolute values were normalized against mean WT values. T: $0.05 < p < 0.1$, *$p < 0.05$.
