# Supplementary figures and images for: Inactivity of Peptidase ClpP Causes Primary Accumulation of Mitochondrial Disaggregase ClpX with Its Interacting Nucleoid Proteins, and of mtDNA
Source: Cells. 2021 Nov 29;10(12):3354. doi: 10.3390/cells10123354 (PMC8699119; doi:10.3390/cells10123354)

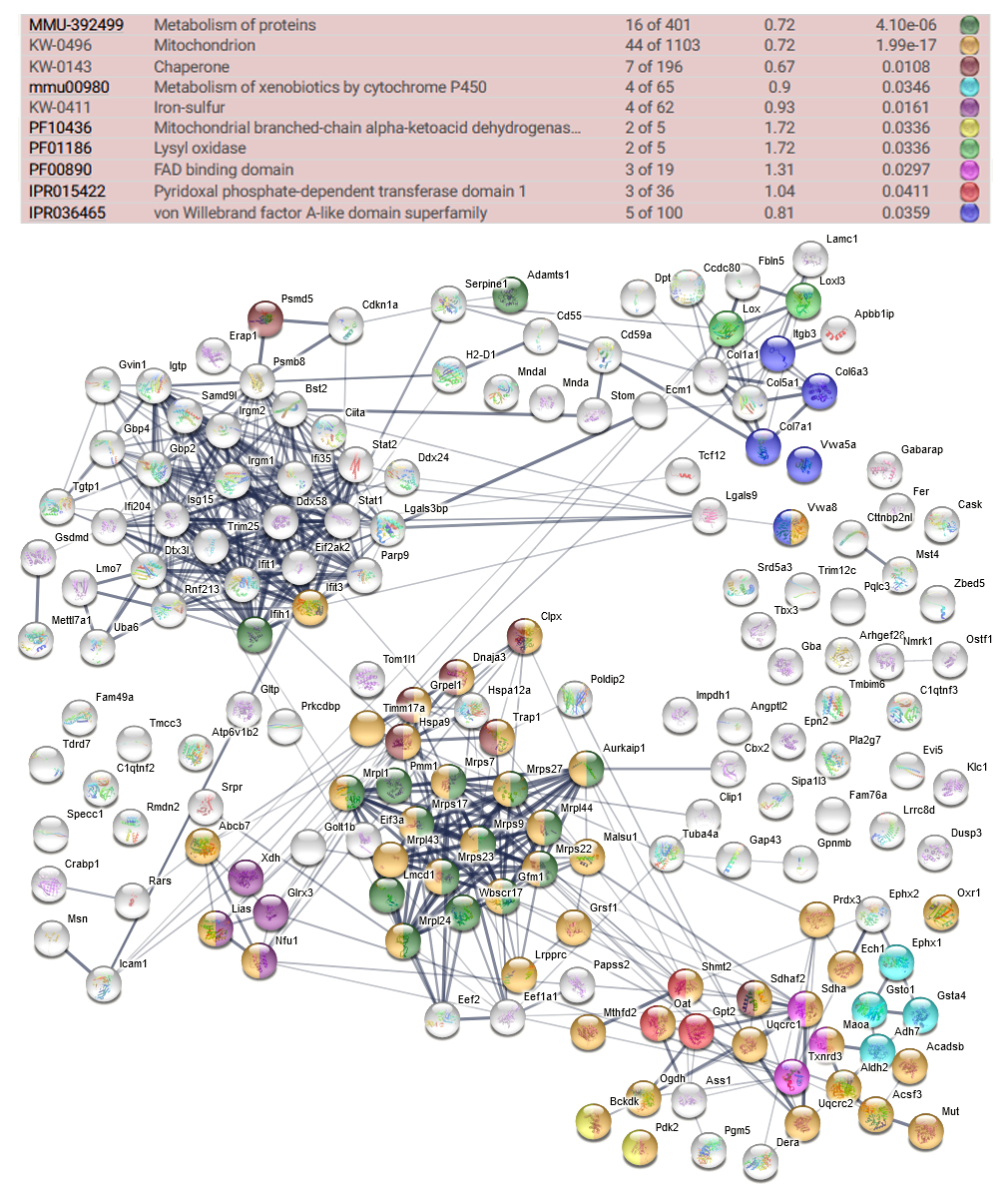

Supplement: Supplementary file 1 [file cells-10-03354-s001.zip › cells-1469176-supplementary resubmitted-final/KeyAuburger-FigureS1_flat.tif]

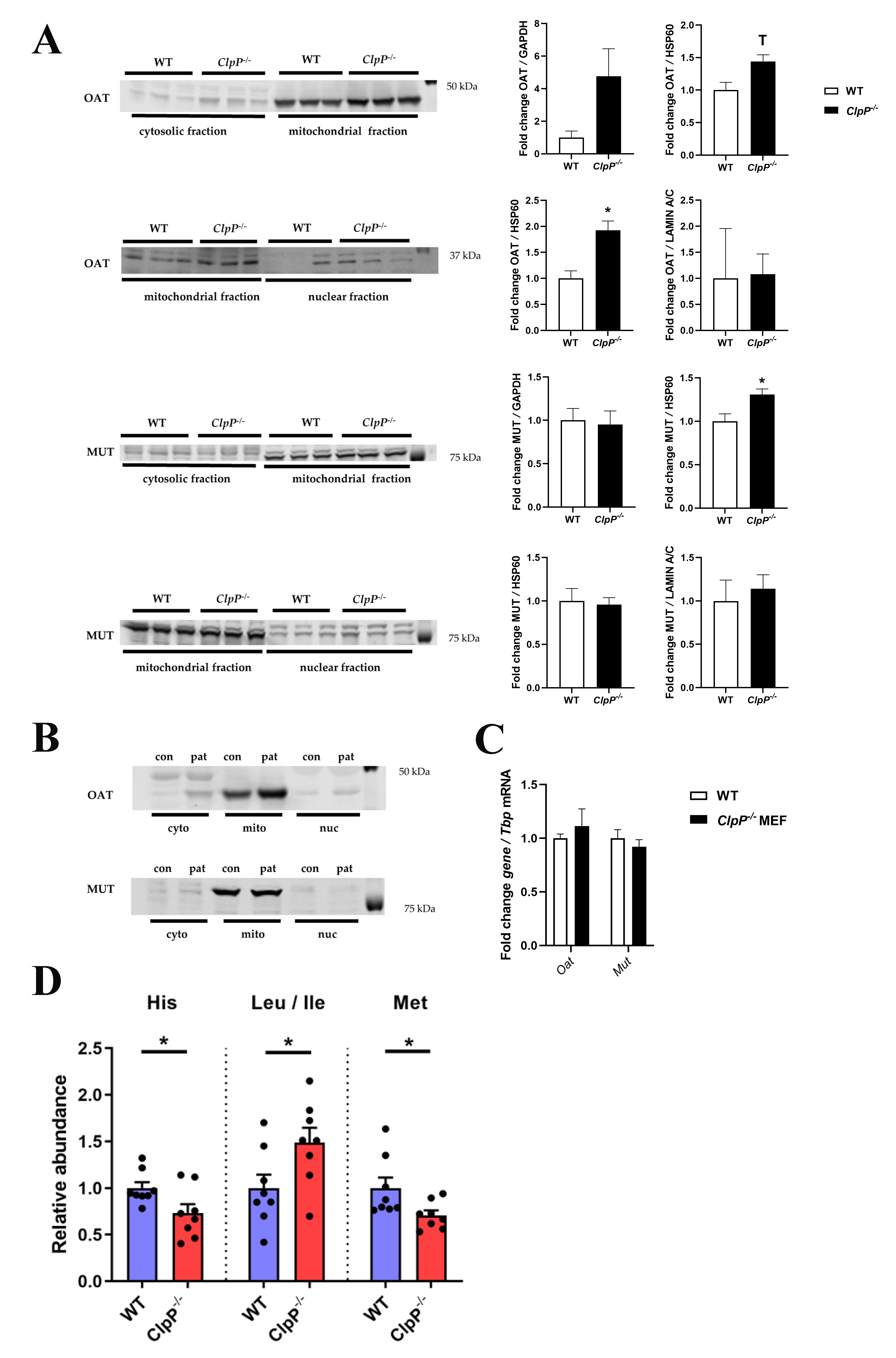

Supplement: Supplementary file 1 [file cells-10-03354-s001.zip › cells-1469176-supplementary resubmitted-final/KeyAuburger-FigureS2_flat.tif]
